# Photopolymerized Mixed Matrix Membranes for Liquid Organic Hydrogen Carrier Separation

**DOI:** 10.1002/advs.202511336

**Published:** 2025-08-28

**Authors:** Abdollah Khosravanian, Farnaz Zadehahmadi, Mohammed Nizam Khan, Hamidreza Mahdavi, Michael T. Scalzo, Declan McNamara, Benny D. Freeman, Matthew R. Hill, Timothy F. Scott

**Affiliations:** ^1^ Department of Chemical and Biological Engineering Monash University Clayton Victoria 3800 Australia; ^2^ Department of Materials Science and Engineering Monash University Clayton Victoria 3800 Australia; ^3^ John J. McKetta Jr. Department of Chemical Engineering The University of Texas at Austin Austin TX 78712 USA

**Keywords:** hydrogen transport, mixed matrix membranes, organic solvent nanofiltration, photopolymerization

## Abstract

Liquid organic hydrogen carriers (LOHCs) are infrastructure‐compatible media for hydrogen storage and transport under ambient conditions, addressing hydrogen's volatility, low density, and high reactivity. Separating liquid hydrogen‐lean/hydrogen‐rich hydrocarbons without resorting to energy‐intensive phase changes is a key barrier to LOHC system implementation. Membrane operations that can separate hydrogen‐lean/hydrogen‐rich species can drive equilibria of dehydrogenation processes, enabling them to run at lower temperatures. Here, new photopolymerized mixed‐matrix membranes composed of a cyclic monomer and a metal organic framework (MOF)/palladium‐doped activated carbon mixture are presented. By leveraging an in situ photopolymerization strategy, high–filler‐loaded mixed‐matrix membranes exhibiting ideal aromatic/aliphatic selectivities of ≈12 and a toluene/methylcyclohexane (MCH) separation factor of 1.8 for membranes with filler loading of 20 wt.% are fabricated. This fabrication approach enables high filler loadings in the monomer and, for multi‐layer films, delivers a stratified structure where it induces the formation of multiple internal polymeric dense “skin” layers, which is ideal for the promotion of selective transport. It is compared that the early studies reported using only commonly reported metrics, rejection versus mixture permeance, with the present system, demonstrating the highest performance to date. This work highlights a high‐performance solvent nanofiltration platform for LOHC separation aligned with sustainable hydrogen production goals.

## Graphical abstract

Photopolymerized membranes with high metal organic framework (MOF)/Pd activated carbon loading enable selective separation of hydrogen‐rich/lean liquid organic hydrogen carriers (LOHCs) under mild operational conditions. The employed fabrication approach yields stratified, multilayer structures with enhanced aromatic/aliphatic (toluene/methylcyclohexane) selectivity. This solvent nanofiltration platform advances energy‐efficient LOHC purification, which is aligned with sustainable hydrogen transportation.

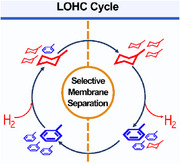

## 1 Introduction

**Figure 1:**
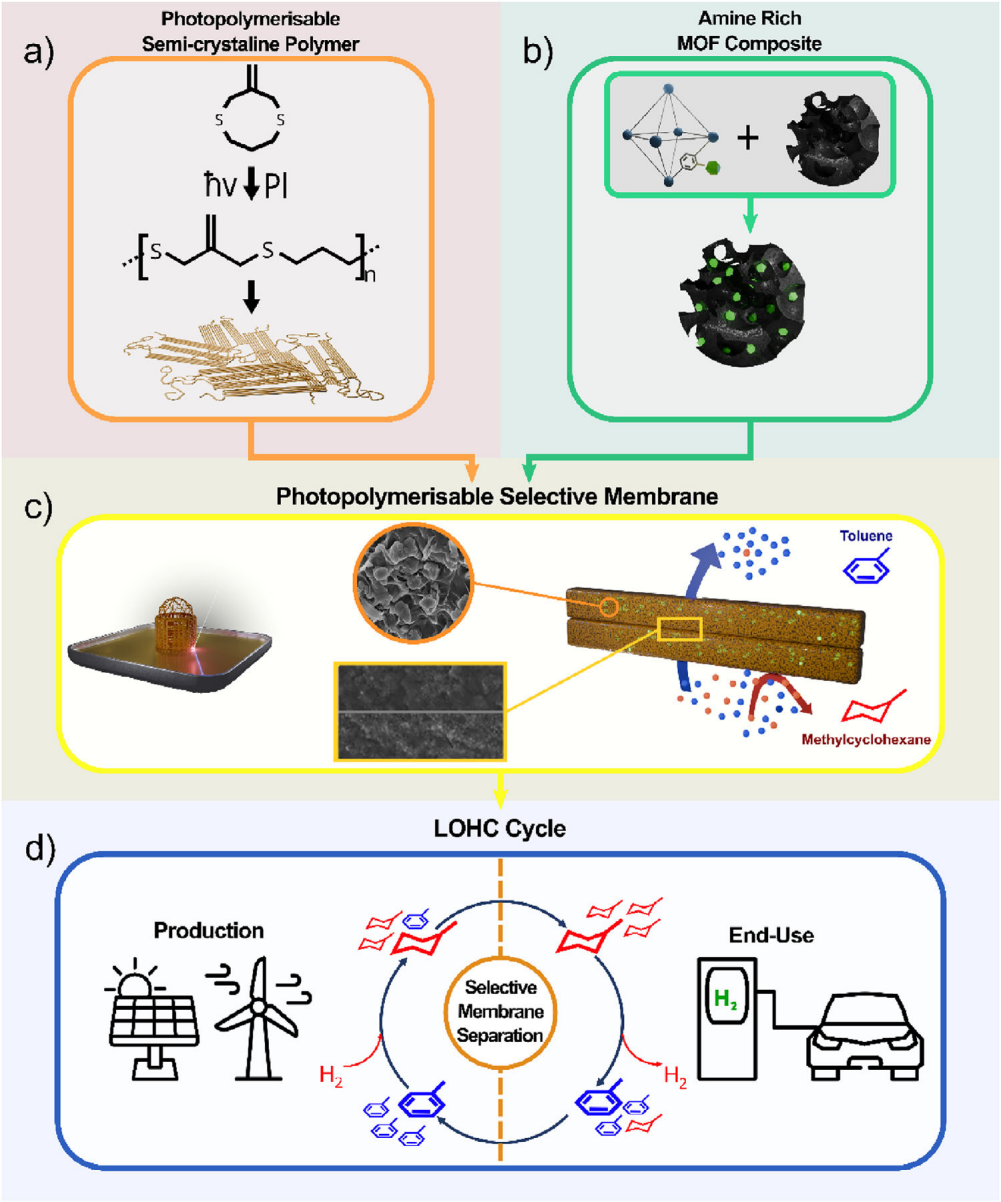
Design and fabrication of novel MMMs for LOHCs utilization. a) CAS8 photopolymerization, b) synthetic route for the preparation of the composite UiO‐66‐NH_2_/PdAC, and c) CAS/UiO‐66‐NH_2_/PdAC membrane fabrication for toluene/MCH separation, and d) schematic illustration of hydrogen transportation using toluene/MCH pairs by existing infrastructure.

Hydrogen has long been recognized as an energy storage medium with the potential to facilitate energy system decarbonization.^[^ ^1^, ^2^, ^3^, ^4^, ^5^, ^6^ ^]^ Nevertheless, challenges associated with transporting molecular hydrogen in bulk remain unresolved, including safety concerns associated with its flammability, the energy demand of compression and liquefaction processes required for transport at volume, and boil‐off losses.^[^ ^7^, ^8^, ^9^, ^10^ ^]^ A commonly proposed approach to address hydrogen transportation issues is to circumvent them by employing molecular hydrogen carrier species such as metal hydrides or ammonia. One class of such hydrogen carriers is that of liquid organic hydrogen carriers (LOHCs), materials that provide a convenient means for storing and transporting hydrogen at scale as they remain liquid under a broad environmental range.^[^ ^11^, ^12^, ^13^, ^14^ ^]^ Moreover, high‐level assessments have established that LOHCs could enable the reutilization of the vast existing infrastructure already established for petroleum storage and transportation^[^ ^15^, ^16^, ^17^, ^18^ ^]^ (**Figure** **1**a).

Several LOHCs pairs with advanced technology readiness levels (TRLs) have been assessed, typically either homocyclic‐based pairs such as dibenzyltoluene/perhydro‐dibenzyltoluene, or heterocyclic compounds including quinoline and indole derivatives.^[^ ^19^, ^20^ ^]^ In particular, the toluene/methylcyclohexane (MCH) pair is a mature exemplar owing to its established reaction pathways, relatively stable cycling, and good hydrogen storage capacity, and has been extensively deployed in industrial and pilot‐scale applications.^[^ ^21^ ^]^ Whereas fully hydrogenated toluene (i.e., MCH) can store hydrogen at a specific volumetric density of 46 kg H_2_ m^−3^, approximately two thirds of the hydrogen per unit volume of liquid hydrogen,^[^ ^22^ ^]^ it does not suffer from the intractable challenges associated with liquid hydrogen production and transportation.

The generic processing of LOHCs involves two fundamental reaction steps: hydrogenation and dehydrogenation. These processes are limited by thermodynamics, where incomplete reaction can occur if the equilibrium is insufficiently shifted toward the product(s) under the employed conditions, resulting in a fraction of the transported liquid being effectively inactive. Moreover, insufficient catalyst selectivity for the dehydrogenation reaction can generate side products that are unavailable for hydrogen carrying in future transportation cycles.^[^ ^23^, ^24^ ^]^ By employing reaction conditions that limit side product formation, process yields could be improved by implementing a post‐reactor separation of the LOHC hydrogen‐rich and hydrogen‐lean forms in conjunction with a recycle stream enriched with the hydrogen‐rich form.^[^ ^19^, ^25^, ^26^ ^]^

Separating hydrogen‐lean aromatics from their corresponding hydrogen‐rich aliphatic forms is a non‐trivial task; indeed, these separations have been described as among the most difficult to perform,^[^ ^27^ ^]^ requiring multi‐process techniques such as extractive distillation using ionic liquids or solvents as extractants.^[^ ^28^, ^29^, ^30^ ^]^ The challenges associated with these separations are ultimately attributable to the similar molecular sizes and small differences in boiling points of the mixture constituents. As the separation of hydrocarbons is most commonly achieved in the petrochemical sector by energy‐ and capital‐intensive fractional distillation, the development of separation techniques that do not require a phase change, thereby precluding the concomitant high energy cost, has been highlighted as an ongoing research area.^[^ ^31^, ^32^ ^]^

Hydrocarbon separation using membranes offers substantial financial and environmental advantages. While membrane‐based separations require appropriate design strategies tailored to the membrane type and the specific separation target, the associated costs have been shown to be significantly lower than those of conventional distillation.^[^ ^33^ ^]^ For example, Marchetti et al. reported that to concentrate 1 m^[^ ^3^ ^]^ of methanol by a factor of 10, the energy required for separation using distillation and membranes is 1750 and only 3 MJ, respectively. This further indicates the huge gap in energy consumption between distillation and membrane filtration.^[^ ^34^ ^]^ Particulate porous materials, including MOFs,^[^ ^35^ ^]^ zeolites,^[^ ^36^ ^]^ silica,^[^ ^37^ ^]^ and other porous structures^[^ ^27^, ^38^, ^39^ ^]^ have shown significant potential for the selective adsorption of aromatic compounds over their aliphatic counterparts; however, the practical application of these materials in large‐scale organic solvent nanofiltration (OSN)‐type separations necessitates their integration into a process‐compatible format, such as within mixed‐matrix membranes (MMMs).

MMMs, commonly composed of particulate fillers dispersed in a continuous polymeric matrix, are promising composite materials for challenging fluid separation processes.^[^ ^40^, ^41^, ^42^ ^]^ The separation performance of an MMM is often determined by the filler chemistry, morphology, and loading, while the polymer phase provides the mechanical integrity necessary for utilization.^[^ ^43^ ^]^ A wide range of polymers has been employed as the continuous matrix in MMMs; nevertheless, for liquid separations, the membrane performance is strongly dependent on the compatibility of the polymer with the process mixture.^[^ ^41^, ^44^ ^]^ Amorphous polymers tend to swell or dissolve upon exposure to organic solvents, limiting their utility in such systems.^[^ ^45^, ^46^ ^]^ In contrast, semi‐crystalline polymers can offer improved liquid phase compatibility, often resisting swelling and dissolution in a wide range of organic solvents.^[^ ^44^, ^47^, ^48^ ^]^

The fabrication of MMMs commonly proceeds via the casting of a particulate suspension in a polymer solution and subsequent solvent evaporation, enabling film solidification and encapsulation of the filler particles.^[^ ^49^, ^50^ ^]^ Owing to the difficulty of achieving homogeneous particle dispersion in polymer melts, the solution casting fabrication approach is employed to afford relatively high filler loadings, desirable given the typical filler‐dependent selectivity, but still faces several challenges, including non‐uniform filler dispersion,^[^ ^51^ ^]^ structural defects, limited mechanical strength,^[^ ^52^ ^]^ and difficulties in increasing filler loading. As an alternative to this casting approach, several studies have described the use of a monomeric carrier for the filler, which can subsequently be polymerized in situ, enabling high filler loadings owing to the relatively lower viscosities of monomer‐filler blends.^[^ ^53^, ^54^, ^55^, ^56^ ^]^ Of the few liquid monomers that provide semi‐crystalline polymers upon polymerization under ambient conditions,^[^ ^48^, ^57^, ^58^, ^59^ ^]^ cyclic allyl sulfides (CAS) present a class of low viscosity liquid monomers that readily participate in radical‐mediated polymerization (Figure 1b) capable of affording semi‐crystalline polymers that exhibit negligible swelling or solubility in most organic solvents.^[^ ^60^, ^61^, ^62^ ^]^ They can also be photopolymerized at room temperature, thereby precluding potential degradation of the selected filler at raised temperatures.

In this work, we describe the successful utilization of photopolymerization to fabricate, at room temperature, robust and free‐standing hybrid MOF/activated carbon (AC)‐based MMMs capable of liquid‐phase toluene/MCH separation (Figure 1c,d). The MOF/AC blend demonstrated both aromatic/aliphatic selectivity as well as excellent compatibility with the employed carrier monomer. Owing to the low monomer viscosity, up to 30 wt.% of the MOF/AC could be readily and homogeneously incorporated into the monomer to afford a photopolymerizable composite resin, enabling the fabrication of membranes with predetermined thicknesses in a single step. By replacing AC with Pd‐doped AC in the prepared membrane, separation factors of up to 1.8 for toluene/MCH mixtures were achieved using a multi‐stage (although single‐process) separation conducted at ambient temperature.

## 2 Results

### 2.1 Rational Design of MOF/Pd‐Activated Carbon MMMs

UiO‐66, representative of zirconium‐based MOF materials, was initially used as the particulate filler owing to its excellent stability and robustness^[^ ^63^ ^]^ as well as its demonstrated differential vapor phase affinity for toluene over MCH.^[^ ^35^ ^]^ Unfortunately, at raised particle loadings, UiO‐66 exhibited a tendency to agglomerate when mixed with the CAS8 monomer, resulting in the generation of inhomogeneous films upon polymerization (Figure S1a, Supporting Information). In contrast, blends of an amine‐bearing derivative of UiO‐66 employing 2‐aminoterephthalic acid (aminobenzene dicarboxylic acid, BDC–NH_2_) as the linker, UiO‐66‐NH_2_,^[^ ^64^ ^]^ in CAS8 afforded well‐distributed mixtures which, upon polymerization, yielded homogeneous films (Figure S1c, Supporting Information). Nevertheless, initial transport experiments indicated limited permeate selectivity for these UiO‐66‐NH_2_/CAS8 films. Noting that palladium is capable of preferentially binding with aromatic over aliphatic species via reversible π complexation interactions,^[^ ^65^, ^66^, ^67^ ^]^ we sought to improve the aromatic/aliphatic selectivity of the fabricated films via the incorporation of palladium in the particulate phase. Whereas Pd‐doping of MOFs has been previously achieved by the in situ reduction of Pd^2+^, we opted to dope our materials with Pd on carbon (PdAC) owing to its synthetic reproducibility and accessibility.^[^ ^68^, ^69^, ^70^, ^71^ ^]^ Zeta‐potential measurements revealed that UiO‐66‐NH_2_ was positively surface charged, +2.1 mV, presumably attributable to the NH_2_ groups, while PdAC was negatively surface charged, −21.9 mV, affirming the typically observed negative surface charge of activated carbon, and thus it was expected that these materials would interact strongly with each other.^[^ ^72^ ^]^ Vapor sorption measurements were performed using the UiO‐66‐NH_2_/PdAC particle mixture at 298 K (Figure S7, Supporting Information), confirming that toluene/MCH selectivity was maintained in the mixture.

**Figure 2:**
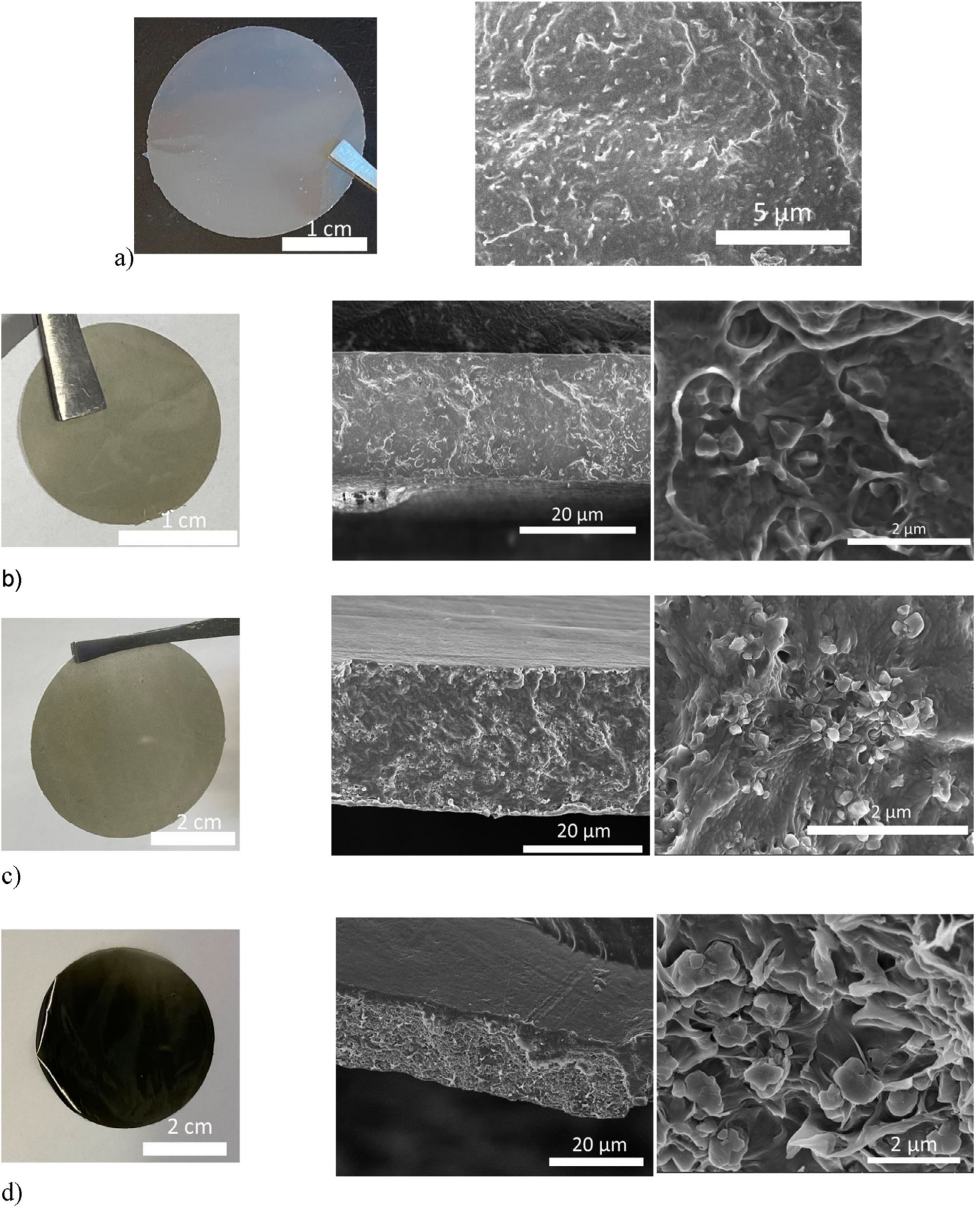
Optical images and scanning electron micrographs of the fabricated membranes. a) Unfilled CAS8. b) 10, c) 20, and d) 30 wt.% of 80/20 UiO‐66‐NH_2_/PdAC in CAS8.

To ensure the compatibility of PdAC with the continuous phase, photopolymerized films were fabricated using formulations of exclusively PdAC blended in CAS8; unfortunately, even at low particle loadings, the fabricated films were inhomogeneous and exhibited visible PdAC agglomerates and through‐film defects (Figure S1b, Supporting Information). This poor filler/matrix compatibility was addressed by mixing PdAC with UiO‐66‐NH_2_ in the solid phase to generate a particulate filler mixture. Subsequent blending of this filler mixture with CAS8 yielded resins with well‐dispersed particles and, upon polymerization, homogeneous films even at high particulate loadings (**Figure** **2**). Notably, limiting the particulate mixing time to less than 5 h yielded defective films owing to MOFs and AC separation and agglomeration during fabrication (Figure S2, Supporting Information).

Fourier transform infrared (FTIR) spectroscopy was employed to determine if any interparticle interactions arose as a result of the MOF/AC mixing process, as well as ensure that the MOF structure was not degraded upon mixing with AC (see Figure S3b, Supporting Information). Whereas the IR spectrum of PdAC revealed little chemical information, the spectrum for UiO‐66‐NH_2_ matched well those described previously for that material,^[^ ^73^, ^74^ ^]^ including peaks at 1256 and 1336 cm^−1^, attributable to C_ar_‐N stretching modes, a peak at 768 cm^−1^, assigned to C–H vibrations in the BDC–NH_2_ benzene ring, bands around 810–690 cm^−1^ attributed to C–H vibration, C–C stretch, OH bend, and OCO bend modes in BDC–NH_2_, a band around 556 cm^−1^ owing to a Zr–(OC) asymmetric stretch, and bands around 657 and 474 cm^−1^ attributed to the µ_3_–O stretch and the µ_3_–OH stretch, respectively.^[^ ^75^ ^]^ With respect to the MOF‐borne amine groups, peaks centered at 3514 and 3400 cm^−1^ are symmetric and asymmetric N–H stretching vibrations, respectively,^[^ ^73^, ^76^ ^]^ while the peak at 1628 cm^−1^ is attributed to an amine bending vibration.^[^ ^77^ ^]^ Interestingly, each peak attributed to an amine vibration mode progressively shifted to a lower wavenumber with raised PdAC content in the particulate mixture, suggesting that the pendant amine groups participate in inter‐particle interactions with the PdAC and enhance dispersion of the particles when added to the monomer formulation.

To further investigate the interparticle interactions between UiO‐66‐NH_2_ and PdAC, X‐ray photoelectron spectroscopy (XPS) and energy‐dispersive X‐ray spectroscopy (EDX) were performed on the particulate mixtures. The N 1s XPS study on PdAC, UiO‐66‐NH_2_, and its composites with PdAC at weight ratios of 80:20 and 50:50 reveals changes in the chemical environment of nitrogen atoms, reflecting the influence of PdAC interactions (Figure S4, Supporting Information). In pristine PdAC, the N1s spectra show no peaks, confirming the absence of nitrogen‐containing groups. In contrast, UiO‐66‐NH_2_ exhibits distinct peaks at binding energies of 397.78 eV for N─C, 398.67 eV for ─NH_2_, and 399.74 eV for ─NH_3_^+^ (Figure S4a, Supporting Information). In the composites with PdAC, these peaks shift to higher binding energies: for the 80:20 composite (UiO‐66‐NH_2_:PdAC), the peaks are observed at 399.01 eV for N─C, 399.81 eV for ─NH_2_, and 400.19 eV for ─NH_3_^+^ (Figure S4b, Supporting Information); and for the 50:50 composite, they shift further to 399.24 eV for N─C, 400.2 eV for ─NH_2_, and 401.52 eV for ─NH_3_^+^ (Figure S4c, Supporting Information). This increase in binding energies suggests a change in the chemical environment of the nitrogen atoms, potentially indicating a decrease in electron density or altered bonding due to interactions with PdAC, rather than an increase in electron density as might be expected from electron donation by Pd. Furthermore, the relative atomic percentages of the nitrogen species vary across the samples. In UiO‐66‐NH_2_, the proportions are 6.02% for N─C, 65.72% for ─NH_2_, and 27.26% for ─NH_3_^+^. In the 80:20 composite, these shift to 7.8% for N─C, 61.86% for ─NH_2_, and 30.34% for ─NH_3_^+^, while in the 50:50 composite, they become 9.01% for N─C, 52.48% for ─NH_2_, and 38.52% for ─NH_3_^+^. These changes demonstrate an increase in the N─C and ─NH_3_^+^ species and a decrease in the ─NH_2_ species with increasing PdAC content. This trend suggests that the interaction with PdAC may reduce the number of free amine groups (‐NH_2_), possibly due to coordination or bonding with Pd, while promoting the formation of protonated (─NH_3_^+^) or differently bonded nitrogen species (N─C).^[^ ^78^, ^79^, ^80^, ^81^ ^]^

The Zr and C EDX mapping of 80/20 UiO‐66‐NH_2_/PdAC mixture reveals the interactions of UiO‐66‐NH_2_ and PdAC in the particulate mixture. As shown in Figure S5 (Supporting Information), the MOF particles appear to reside within the pores of PdAC, facilitating uniform filler distribution in the membranes, a result that was unattainable when PdAC was added without the MOF.

The FT‐IR spectrum of a photopolymerized CAS/UiO‐66‐NH_2_/PdAC film (Figure S3a, Supporting Information) exhibited the characteristic peaks of both CAS8 and UiO‐66‐NH_2_ with no notable wavenumber shifts observed between the unmixed and mixed materials.

The impact of each component in the composite mixture on the resultant microstructure was assessed via XRD (Figure S6, Supporting Information). Here, the UiO‐66‐NH_2_ diffractogram was found to match previously reported diffractograms, with peaks at ≈7.3°, ≈8.5°, and ≈12.0° (2θ) corresponding to diffraction from the (111), (200), and (220) planes, respectively.^[^ ^82^ ^]^ The addition of PdAC to UiO‐66‐NH_2_ was found not to influence the MOF crystal structure. Similarly, the diffractogram of the composite film revealed no influence of the polymer on the particle structure, nor of the particulate mixture on the polymer crystallinity.

### 2.2 Mixed Matrix Membrane Fabrication

Composite resins for the fabrication of mixed matrix membranes (MMMs) via ring‐opening photopolymerization were prepared using the UiO‐66‐NH_2_/PdAC particle mixture in conjunction with the cyclic allyl sulfide monomer CAS8 and the photoinitiator BAPO. Before membrane fabrication, the rheological behavior of the composite CAS8/UiO‐66‐NH_2_/PdAC resin was characterized as the resin viscosity, photopolymerization rate, and crystallization behavior impact the membrane thickness and microstructure.

**Figure 3:**
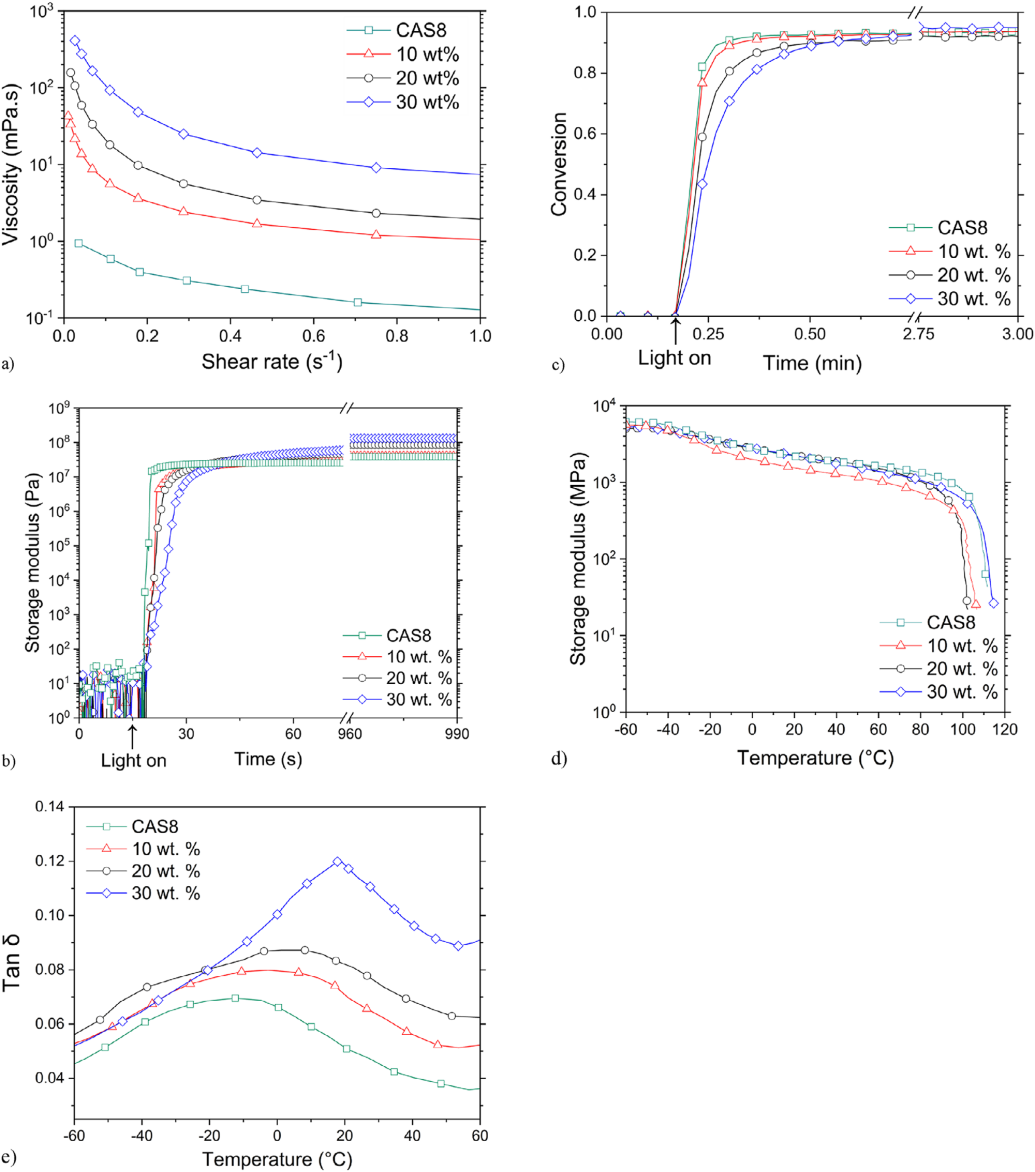
Characterization of unfilled and 80/20 UiO‐66‐NH_2_/PdAC‐filled CAS8 resin formulations. a) Viscosity versus shear rate for unfilled and filled CAS8 at 25 °C. b) Storage modulus trajectories, determined with photorheometry using a 50 µm gap, 12 mm diameter parallel plate geometry, for unfilled and filled CAS8 resin formulations during irradiation with 400–500 nm light at an intensity of 3.4 mW cm^−2^. c) Monomer conversion trajectories, determined with FTIR spectroscopy using a collection rate of ≈2 scans/second and monitoring the disappearance of the peak centered at 830 cm^−1^ for unfilled and filled CAS8 resin formulations during irradiation with 365 nm light at an intensity of 20 mW cm^−2^. d) Storage modulus and e) tan δ curves of photopolymerized, unfilled, and filled CAS8 samples, determined with DMA using a temperature ramp of 5 °C min^−1^ at a strain of 0.1% and frequency of 1 Hz.

During membrane fabrication, the actuated printer build platform is moved down to a predetermined distance from the projection window, dictated by the intended thickness of the resultant film, imposing a shear on the composite resin. Whereas the incorporation of high filler loadings in mixed matrix membranes is a desired goal, the printability of a resin is in part determined by its ability to flow during build platform movement, which in turn is a function of the resin viscosity. Thus, viscosity as a function of shear rate was characterized for resins incorporating a range of filler loadings. The viscosity of the composite decreased with raised shear rate, regardless of filler loading (**Figure** **3**a), indicating shear‐thinning behavior typical of slurries.^[^ ^83^ ^]^ The optimal resin viscosity for SLA 3D printing at low shear rates is reported to be under 5 Pa·s.^[^ ^83^, ^84^ ^]^ For filler loadings of 0% and 10%, the viscosity remained well below 100 mPa·s for all shear rates examined, and only slightly exceeded 100 mPa·s for a 20 wt.% filler loading resin at very low shear rates (Figure 3a; Figure S8, Supporting Information). At a filler loading of 30 wt.%, the zero shear viscosity was found to be ≈500 mPa·s (Figure 3a), allowing films to be readily fabricated under ambient conditions at this filler loading; however, the resin viscosity rapidly increased with raised filler loadings beyond 30 wt.%, precluding facile film fabrication.

Solvent/polymer compatibility is a key factor in OSN, such that the development of materials with broad solvent compatibility offers significant value. In addition to their low viscosity and capacity for photopolymerization under ambient conditions, the solvent compatibility and mechanical integrity of the resultant polymers make unfunctionalized cyclic allyl sulfide monomers such as CAS8 well‐suited for OSN membrane fabrication. Whereas poly(CAS8) is a highly crystalline thermoplastic polymer with negligible solubility or swelling in many organic solvents,^[^ ^48^ ^]^ including toluene and MCH, the cyclic allyl sulfide monomer CAS8 is miscible in all proportions with most organic solvents. Consequently, to minimize the leachable content and ensure the solvent resistance and integrity of the polymerized films upon exposure to toluene and MCH, the polymerization and crystallization kinetics of both unfilled and filled CAS8 resin formulations were examined using photochemistry (Figure 3b). Upon exposure to blue light, the storage modulus for the unfilled CAS8 formulation increased rapidly, approaching a plateau after ≈15 seconds of irradiation, while the addition of the particle mixture filler in increasing amounts to the CAS8 formulation was accompanied by an apparent progressive reduction in modulus development rate. In a previous study, the rapid modulus development during the photopolymerization of CAS8 was attributable to polymerization and crystallization proceeding concurrently, whereas for systems where the polymer crystallization was delayed and proceeded after the initial photopolymerization, a distinct shoulder was observed in the storage modulus development curves.^[^ ^48^ ^]^ Similar, although slight, shoulders are observed in the curves for UiO‐66‐NH_2_/PdAC‐filled CAS8 shown here, suggesting that the filler addition may impede the polymerization and/or crystallization process.

Notably, any light intensity gradient through the 50 µm sample thickness, a potential consequence of light scattering from non‐index matching between the monomer and filler particles as well as the strong visible light absorption by the carbon component of the particle mixture, would also be reflected in the modulus development and conversion curves as the volume closest to the light source would react more quickly than that farthest away from the source. UV–visible spectrophotometry was performed on both unfilled and filled resin formulations to determine if a light intensity gradient through the sample thickness might impact the observed photopolymerization behavior. As shown in Figure S9 (Supporting Information), a marked increase is observed in blue light absorbance at raised filled loadings, indicating that, despite the relatively short 50 µm pathlength, a significant light intensity gradient is established through the sample during polymerization. Thus, whereas the photorheometry data holds utility for determining photopolymerization parameters, it does not necessarily reveal any fundamental insight into polymerization or crystallization rates.

To confirm the photopolymerization kinetics, FTIR spectroscopy was used to monitor the reaction progress of CAS/UiO‐66‐NH_2_/PdAC resin formulations during irradiation (Figure 3c; Figure S11a–c, Supporting Information). Whereas the pathlength used for the rheometer (i.e., the 50 µm parallel plate gap), necessary to accommodate the monomer‐borne particles and obtain reasonable modulus trajectories, resulted in a non‐trivial light intensity gradient, the pathlength used for FTIR spectroscopic studies was substantially shorter (<10 µm) than that used for the rheometer, alleviating any light intensity gradient impact. Upon sample irradiation, the infrared spectra evolved where peaks at 839 and 1314 cm^−1^ diminished while peaks at 830 and 1306 cm^−1^ concurrently emerged. Given the partial overlap of these peaks, deconvolution via Gaussian peak fitting was employed to determine monomer conversion trajectories. The conversion trajectories indicated rapid photopolymerization, with near‐full conversion achieved for the unfilled resin formulation within a minute of irradiation; however, increased filler loadings were accompanied by decreased polymerization rates, which, given the very short pathlengths employed, could not be attributed to attenuation of the light through the sample thickness. Notably, as the MOF component of the filler mixture being employed bears a pendant amine group, the incorporation of filler into the resin introduces amines into the composite formulations. Cook^[^ ^85^ ^]^ found that the maximum polymerization rate for a radical‐mediated dimethacrylate photopolymerization was reduced at high amine concentrations and concluded that this was because amines tend to retard free radical polymerization at high concentrations.^[^ ^86^ ^]^ Imoto et al.^[^ ^87^ ^]^ similarly described retardation or inhibition of vinyl acetate polymerization upon addition of substituted aniline derivatives. Hoyle and Kim^[^ ^88^ ^]^ found that radical‐mediated diacrylate photopolymerization was increasingly retarded by aromatic tertiary amines as the amine concentration was increased, which they attributed to chain transfer from the propagating radical to the amine, forming an aminyl radical; whereas this radical reinitiates the polymerization, this reinitiation is slower than the normal rate of propagation. If a similar chain transfer effect were occurring here, it would account for the photopolymerization rate retardation observed with raised filler loading and would result in covalent bond generation between the amine‐bearing MOF particles and the polymeric continuous phase.

Figure 3d,e presents storage modulus and tan δ curves, respectively, during temperature ramps applied to unfilled and filled photopolymerized samples. The lower temperature for these temperature ramps was set to be below the glass transition temperature (*T*_g_) of CAS8, while the upper temperature was set to approximate the CAS8 melting temperature to avoid polymer melt flowing into the instrument drive shaft. Whereas incorporation of filler into CAS8 had minimal impact on the storage modulus, the peak in the tan δ curve attributable to the glass transition was found to increase from ≈−10 °C for unfilled CAS8 to 20 °C for CAS8 filled with 30 wt.% UiO‐66‐NH_2_/PdAC. The increase in *T*_g_ from −10 to 20 °C suggests strong filler‐polymer interactions, leading to restricted polymer chain mobility. This indicates good compatibility between UiO‐66‐NH_2_/PdAC and CAS8, likely due to interfacial interactions. These interactions limit segmental motion, increasing the energy required for the polymer to transition to a rubbery state. As a result, the filler is well‐integrated into the polymer matrix, enhancing the composite's thermal properties.^[^ ^89^, ^90^ ^]^

### 2.3 Membrane Fabrication and Characterization

Membrane films were fabricated from photopolymerizable resins formulated from CAS8 filled with the UiO‐66‐NH_2_/ PdAC mixture, as well as a photoinitiator active in the near‐UV/visible region of the spectrum. Using an irradiation wavelength of 385 nm, the maximum attainable thickness of a single‐layer photopolymerized film was ≈35 µm owing to the strong near‐UV absorbance by the carbon component of the filler mixture; consequently, thicker films were obtained by multi‐layer fabrication. Using this photopolymerization system, free‐standing, mechanically robust, pinhole defect‐free mixed matrix membranes were obtained even at 30 wt.% filler, the highest loading examined. Whereas fracture surface SEM images of photopolymerized UiO‐66‐NH_2_/PdAC‐filled CAS8 mixed matrix membranes, as shown in **Figure** **4**a,b (double and single layer, respectively), demonstrated homogeneous filler dispersion in the bulk material, a filler‐depleted skin layer of ≈2.5 µm thick was apparent for all filled samples. To understand why this happens, we conducted an energy dispersive X‐ray analysis (EDX) analysis on the membranes (Figure 4a,b). This analysis looked at the filler distribution using Zr and Pd average counts and EDX mapping. It also assessed the polymer distribution using S average counts and EDX mapping. The analysis revealed a higher density of sulfur on the surface and between the two layers compared to zirconium. This indicates the presence of a skin layer primarily made up of a polymer. This skin layer is highly resistant to solvent permeation, which increases membrane resistance and reduces permeability. However, the presence of PdAC homogeneously dispersed in the membrane confirms the selectivity observed for the prepared membranes. Figure 4a shows that the multistep 3D printing process resulted in a concentration of the polymer relative to the MOF additive at the interfacial surface. This region of increased polymer density is akin to an internal skin layer, offering a novel method to deliver a new thin film composite architecture, where these thin but selective layers are internally supported by the higher porosity, higher diffusion rate section within the membrane. This can deliver enhanced selectivity within a free‐standing polymer. Atomic force microscopy (AFM) was performed to study the surface roughness of the fabricated membranes, revealing progressively increased roughness at raised filler loadings (Figure S11, Supporting Information).

Figure 4Membrane performance and elemental mapping coupled with EDX line scan. a) and b) SEM images and EDX analysis of single and double‐layered 30% CAS/UiO‐66‐NH_2_/AC membranes. c) Permeability versus thickness for membranes with 20 and 30 wt.% filler loading, d) long‐term toluene permeation test for single‐layer membranes with 20 wt.% filler loading. Error for pure toluene permeation was determined to be ≈2.5%–7%, e,f) permeation and separation factor versus toluene/MCH molar ratio for 20 and 30 wt.% membranes, respectively, calculated rejection error for 20 wt.% UiO‐66‐NH_2_/PdAC for 95/5 molar ratios of toluene/MCH was determined to be ≈0.3%–4% and g) ideal versus real selectivity (various feed compositions) comparison for membranes with 20 wt.% and 30 wt.% filler loading.

**Figure:**
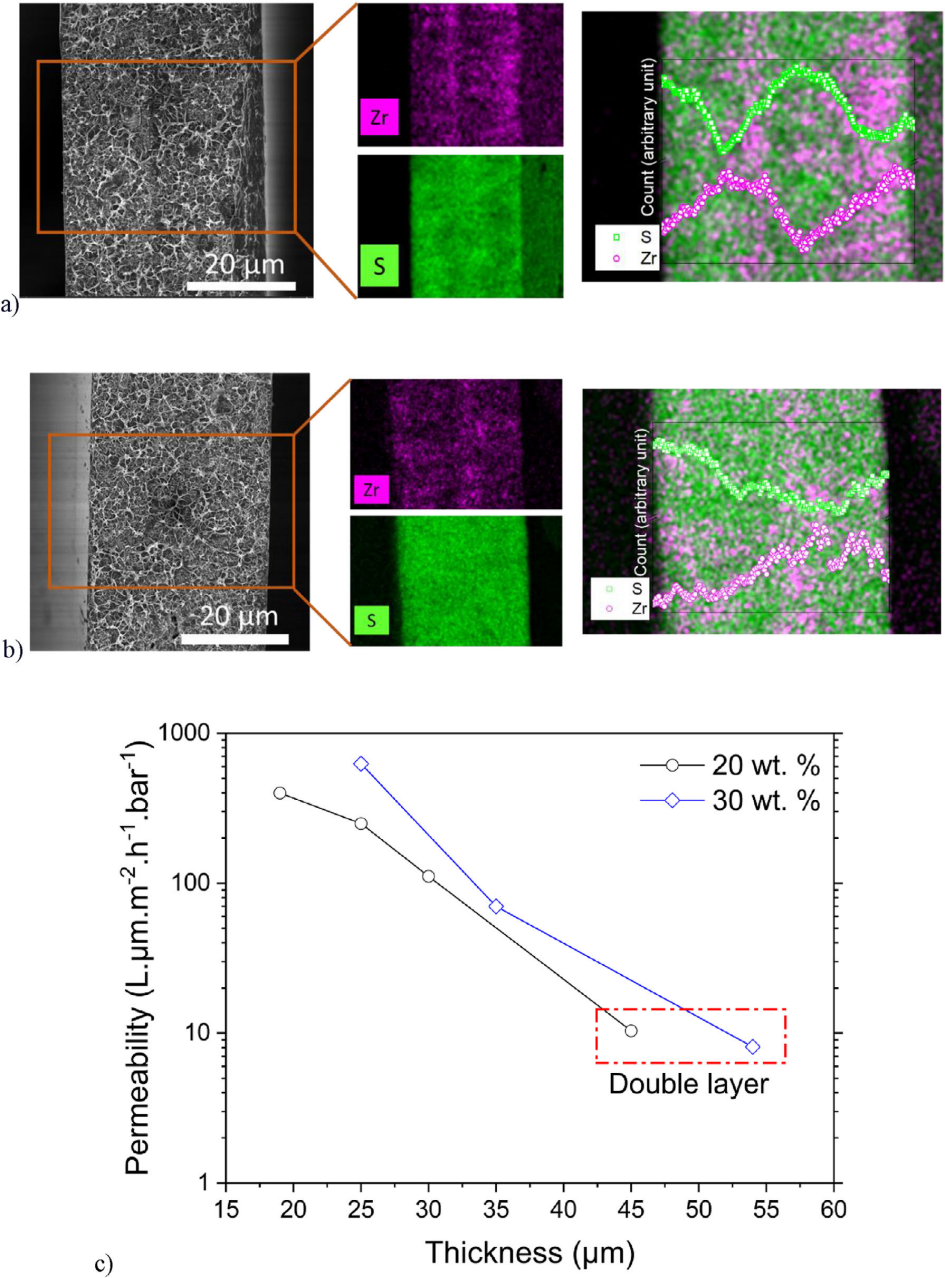

**Figure:**
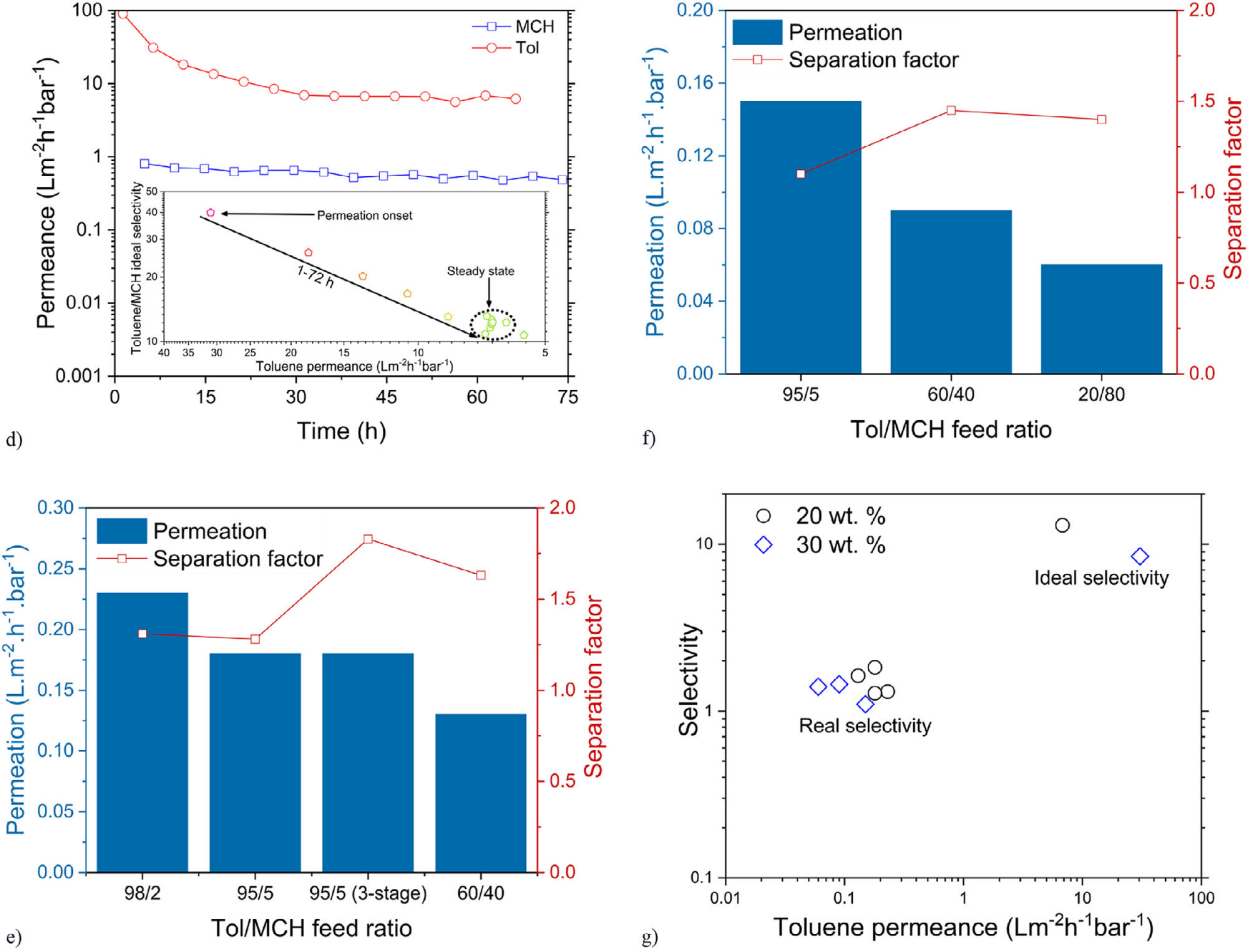

Membrane coupons for liquid‐phase permeance characterization were cut from larger films using a 47 mm diameter circular die and soaked in toluene overnight before testing in a dead‐end cell. Notably, membranes fabricated from unfilled and 10 wt.% particle filled CAS8 showed no toluene permeation over at least 24 h suggesting that, for filler loadings that exceed a threshold value, permeation of solvent molecules proceeds either through the filler pores or potentially through interstitial gaps between the filler particles and polymer matrix, the occurrence of which was not observed and may not be supported if covalent interactions between the MOF‐borne amine groups and polymer matrix arise during the radical‐mediated polymerization. An examination of the impact of thickness on membrane permeability revealed that the permeability decreases with increased film thickness (Figure 4c). Multilayer films were fabricated to further minimize selectivity loss caused by potential filler/polymer interstitial gaps; however, this resulted in a significant decrease in membrane permeability owing to the formation of slow‐permeance, polymer‐rich skin layers on the membrane surfaces and between the layers (Figure 4c). The membrane permeation results through 20 wt.% UiO‐66‐NH_2_/PdAC‐filled CAS8 for single‐component solvents demonstrates a consistently higher permeation rate (Figure 4d), thus, higher ideal selectivity for toluene than that for MCH, indicating preferential toluene transport over MCH attributable to UiO‐66‐NH_2_ and PdAC selective affinity for toluene.^[^ ^64^, ^91^ ^]^

Given the ideal selectivity for toluene over MCH, suggestive of a capacity for selective separation in a mixture, toluene/MCH mixtures with different molar ratios were used as feed to analyze permeation and separation performance of the membranes (Figure 4e,f). Here, the membrane performance was evaluated using a cross‐flow filtration setup after 24 h of permeation of the feed solvent. Employing proton nuclear magnetic resonance (^1^H‐NMR) to analyze the ratio of solvent components in the permeate (Figures S12–S17, Supporting Information), Figure 4e illustrates the permeation and separation factor for membranes with 20 wt.% filler loading and a thickness of 45 µm. The permeation of the mixed solvent through the membrane decreased as the molar ratio of MCH in the feed increased from 0.02 to 0.4, indicating faster permeation of toluene through the membrane. The separation factor for toluene/MCH feed ratios of 98/2 and 95/5 is around 1.3, and as the MCH molar ratio is increased to 0.4, the membrane shows increased selectivity for toluene, with the separation factor reaching 1.6. The 95/5 toluene/MCH ratio was tested in a three‐stage separation process, and as expected, the separation factor for the 3‐stage separation increased to 1.83. The observed selectivity for toluene over MCH in the studied mixed solvent may be attributable to the interaction of non‐polar aromatic volatile organic compounds (VOCs), such as toluene, with the aromatic terephthalate linkers in UiO‐66‐NH_2_ through π–π stacking.^[^ ^92^ ^]^ Additionally, the amine groups in the MOF can interact with toluene's π‐electron cloud.^[^ ^93^ ^]^ These combined effects improve the preferential adsorption of toluene within the membrane matrix compared to non‐aromatic compounds like MCH, which cannot participate in such interactions.^[^ ^92^, ^93^ ^]^ Regarding the Pd role in increasing the selectivity, to the best of our knowledge, no paper has discussed the mechanistic routes in detail. However, Studies on Pd‐supported catalysts, such as Pd/activated carbon, Pd/carbon, as well as Pd impregnated porous materials, have demonstrated that toluene undergoes preferential adsorption and catalytic hydrogenation on Pd surfaces, which is driven by π‐d interactions and optimized structures. The d‐orbitals of Pd interact with the aromatic ring of toluene through π‐complexation, leading to affinity toward toluene compared to MCH, which lacks π‐electron systems.^[^ ^91^, ^94^, ^95^, ^96^, ^97^, ^98^ ^]^ These findings were further explored by studying the electronic structure of Pd using XPS. The XPS spectrum (Figure S4d, Supporting Information) exhibited two peaks that corresponded to the 3d_3/2_ peaks versus the 3d_5/2_ peaks of Pd. Metallic Pd is represented by the peaks at 332.85 and 341.87 eV, while Pd^2+^ is defined by the peaks at 335.42 and 346.46 eV. The Pd^2+^ and Pd° contents of the PdAC/UiO‐66‐NH_2_ can be determined as 50.2 and 49.8%, respectively, through semiquantitative analysis of the area of each peak.^[^ ^99^ ^]^ Metallic Pd° can form complexes with the conjugated ring of toluene, which increases its sorption affinity.^[^ ^66^, ^100^ ^]^ Additionally, the Lewis‐acidic Pd^2+^ sites preferentially interact with the π‐electron cloud of toluene, potentially enhancing its affinity for toluene relative to MCH.^[^ ^66^ ^]^

Figure 4f shows the permeation and separation factor for the membrane with 30 wt.% filler loading and a thickness of 54 µm. As the filler loading increased, the membrane thickness also increased, leading to lower permeance for membranes with 30 wt.% loading. Similar to membranes with 20 wt.% filler loading, as the MCH content in the feed increased, the permeance decreased, indicating that more MCH slows down the feed permeation. Additionally, as the MCH ratio increased from 0.05 to 0.4, the separation factor increased from 1.1 to 1.45, respectively, and when the MCH ratio was increased to 0.8, the separation factor experienced a slight decrease to 1.41. Compared to 20 wt.%, membranes with 30 wt.% filler loading showed a lower separation factor as well. Therefore, considering the minimum achievable thickness, permeability, and selectivity, a filler loading of 20 wt.% demonstrated better performance for the membranes studied. Although 30 wt.% was still readily processable, the overall performance was lower compared to 20 wt.%, which offered a more favorable trade‐off between processability and separation efficiency. This result reflects a common trade‐off in the fabricated MMMs. Whereas the 30 wt.% composites still maintained processibility and structural integrity upon photopolymerized, the membrane performance was compromised, potentially owing to issues such as filler agglomeration, interfacial defects, or disruption of the polymer matrix continuity. However, the membrane with 30 wt.% loading exhibited slightly higher single‐component solvent permeance, resulting in a marginal improvement in permeance/ideal selectivity performance. Figure 4g shows the comparison between ideal selectivity and real selectivity for membranes with 20 and 30 wt.% filler loading. As it can be seen, the selectivity decreases significantly, which could be due to the complex impact of the mixed solvent as it interacts with the polymer and fillers.

**Figure 5:**
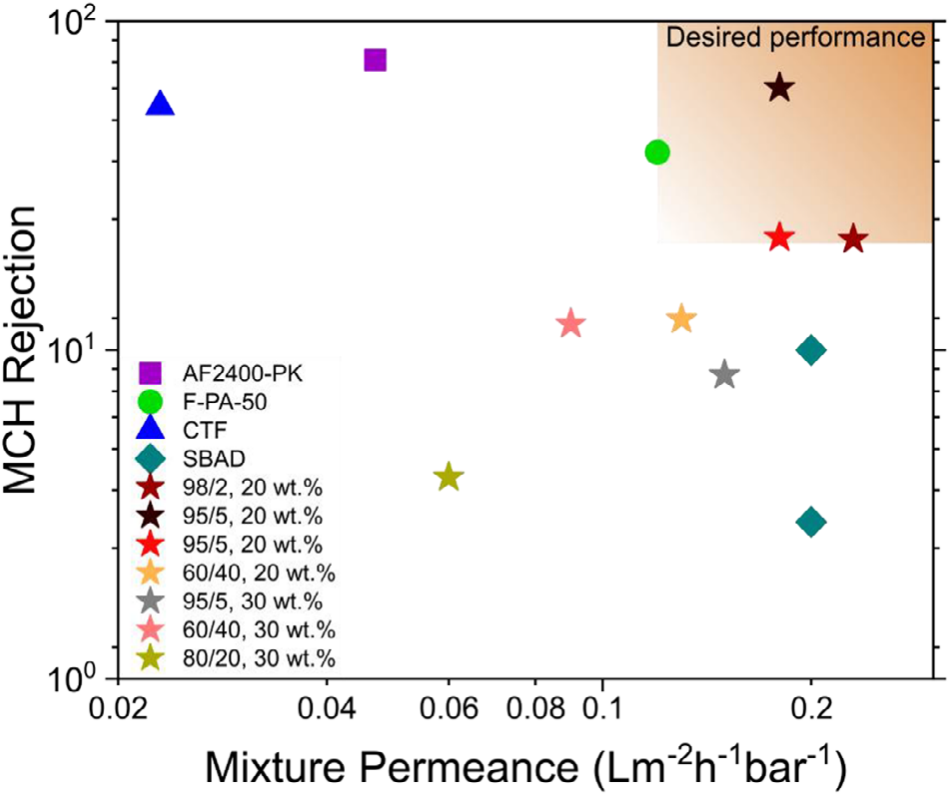
Trade‐off between MCH rejection (%) and solvent mixture permeance (Lm^−2^·h^−1^·bar^−1^) for membranes used in toluene/methylcyclohexane (MCH) separation. N‐aryl–linked spirocyclic polymer‐based membrane (SBAD) from^[^ ^104^ ^]^ with a permeance of 0.2 and MCH rejection of 3% and 10% for membrane module and coupon, respectively, tested at 40 bar and temperature below 200 ⁰C using a cross‐flow system and a toluene rich feed with sampling between 24–66 h of permeation; a covalent triazine framework (CTF) membrane from,^[^ ^101^ ^]^ with a permeance of 0.023 and rejection of 55%, tested at 30 bar using a dead‐end cell and a toluene rich feed with sampling within 12 h; a fluorine‐incorporated thin film composite membranes (F‐PA‐50) from,^[^ ^103^ ^]^ with permeances of 0.12 and MCH rejections of 39.9% using a cross‐flow cell and a toluene rich feed at 30 bar, and a AF2400/polyketone (AF2400‐PK) composite OSRO membrane,^[^ ^102^ ^]^ with permeances of 0.047, and MCH rejections of 76.2%, tested using a cross‐flow cell and a toluene rich feed at 40 bar and 25 ⁰C, where sampling was done within 2 h of permeation. The results from this study, shown as colored stars, were obtained using a cross‐flow cell at 2 bar with sampling within 24 h, and represent membranes with varying filler content and testing conditions: green stars indicate membranes with 20 wt.% filler, while red stars represent those with 30 wt.% filler. Our membranes exhibit a permeability of 0.23 for toluene‐rich feeds and MCH rejection up to 63% across three stages and 22% in a single stage, improving several literature benchmarks and demonstrating the effectiveness of our material design and membrane fabrication technique for aromatic/aliphatic separations.

Of the few papers examining toluene/MCH separation,^[^ ^101^, ^102^, ^103^, ^104^ ^]^ to the best of our knowledge, none have assessed the separation of mixtures with MCH molar fractions over 0.05. Additionally, C_P_/C_R_ (C_P_ and C_R_ are permeate and retentate concentration, respectively) in these studies was either not reported or was under 1.2, while in this study, for 60/40 toluene/MCH molar ratio, C_P_/C_R_ was 1.43 and 1.27 for membranes with 20 and 30 wt.%, respectively. The results in this study were compared to the literature and presented in **Figure** **5**. We compared the early studies reported in this field using the only commonly reported metric, rejection versus mixture permeance, with the present system demonstrating the highest performance to date (Figure 5).

The comparative analysis of membranes with different filler loadings underscores the critical role of microstructural optimization. While increasing filler content can enhance selective transport, excessive loading may lead to particle agglomeration and the formation of structural defects, which negatively impact separation efficiency. Furthermore, the observed disparity between ideal and actual selectivity highlights the non‐ideal behavior of mixed solvent systems and the complex interactions between the polymer matrix, filler particles, and permeating species.

The demonstrated separation performance, particularly under feed conditions not commonly addressed in earlier studies, suggests that the membrane system is well‐suited for handling mixtures from LOHC dehydrogenation processes. In contrast to previous reports, which largely focused on near‐pure aromatic feeds and achieved lower separation factors,^[^ ^101^, ^102^, ^103^, ^104^ ^]^ the present work extends the operating window and offers improved separation performance. The membrane's selective performance, particularly when implemented in a staged configuration, offers a promising strategy for improving the energy efficiency of LOHC systems. By enabling preferential separation of aromatic compounds, improving permeance, and in general moving toward the desired performance area, this approach can become a more economically favorable process while reducing reliance on energy‐intensive thermal processes and supporting the development of more efficient, membrane‐integrated LOHC cycles.^[^ ^105^, ^106^, ^107^ ^]^ The tunability of the membrane through compositional and structural control provides a valuable foundation for further refinement toward scalable, high‐performance separation technologies.

## 3 Conclusion

This study presents a significant advancement in membrane design for LOHCs separation and addresses the persistent challenge of selectively separating toluene and methylcyclohexane to transport hydrogen globally, in a simple manner. The mixed matrix membranes developed here achieved ideal selectivity values exceeding 10 and a real separation factor of 1.8 at the highest‐performing filler loading of 20 wt.% between the loadings studied. The additive manufacturing technique employed afforded high filler loadings (30 wt.%) to be used, which delivered comparatively stable permeation. These results were enabled by the incorporation of a UiO‐66‐NH_2_/PdAC mixture filler, embedded between polymer‐rich dense skin layers. The membrane structure was fabricated using a ring‐opening CAS8 monomer whose favorable storage modulus and viscosity supported robust mechanical performance. Importantly, the membranes were produced via 3D printing using an addition–fragmentation chain transfer mechanism, which enabled the creation of layered, defect‐free architectures. This fabrication method allowed reproducible and tunable fabrication, with consistent performance across varying feed concentrations. While the current study focused on the toluene/MCH system as a model LOHC pair, future investigations involving other promising LOHC pairs, such as dibenzyltoluene/perhydro‐dibenzyltoluene and decalin/naphthalene, are encouraged to assess the broader applicability of membrane design and separation strategy.

## 4 Experimental Section

### Materials

Sodium (cubes, 99.9% trace metals basis), 1,6‐hexanediol, 3‐chloro‐2‐chloromethyl‐1‐propene, methanol, toluene, methylcyclohexane, 10 wt.% loading palladium on carbon (PdAC) and phenyl‐bis(2,4,6‐trimethylbenzoyl)phosphine oxide (BAPO) were purchased from Sigma Aldrich. UiO‐66‐NH_2_ was purchased from Boron Molecular Inc. (Noble Park, Victoria, Australia). All materials were used without further purification.

### Monomer Synthesis

CAS8 was synthesized using a previously reported procedure.^[^ ^62^ ^]^ Briefly, 8.69 g of sodium was added to 1500 mL of methanol under nitrogen in a three‐neck round‐bottom flask equipped with a condenser. After cessation of hydrogen gas evolution (i.e., complete reaction of sodium with methanol), the methanolic sodium solution was brought to reflux and methanolic solutions of 1,3‐propanedithiol and 3‐chloro‐2‐chloromethyl‐1‐propene (20.456 and 23.625 g, respectively, each diluted with methanol to make up 60 mL and transferred into separate syringes) were concurrently added at 3 mL h^−1^ using a syringe pump. Upon complete addition of the precursor solutions, the reaction mixture was refluxed for a further 5 h, cooled, filtered, and the methanol was removed under reduced pressure. The residue was taken up in diethyl ether, extracted using deionized water, and the diethyl ether was removed under reduced pressure. Finally, the residual oil was purified using bulb‐to‐bulb distillation to yield 18.1 g of CAS8 (59% yield, ^1^H NMR (400 MHz, CDCl_3_, 25 °C): δ 1.79 (m, *J* = 6.2 Hz, 2H, SCH_2_CH_2_CH_2_S), 2.88 (t, *J* = 6 Hz, 4H, SCH_2_CH_2_CH_2_S), 3.25 (s, 4H, ═CCH_2_S), 5.19 (s, 2H, ═CH_2_).

### Resin Formulation

To obtain a particulate mixture incorporating 2 wt.% Pd, a palladium loading commonly achieved via in situ Pd^2+^ reduction techniques,^[^ ^108^ ^]^ particle mixtures were prepared by adding 0.5 g of 10 wt.% loading palladium on carbon (PdAC) to 2 g of UiO‐66‐NH_2_ and mixed using a magnetic stirrer for at least 5 h to ensure complete particle commixing. While UiO‐66‐NH_2_/AC composites were synthesized via solvothermal methods,^[^ ^109^ ^]^ a simpler solid‐phase approach was employed to prepare UiO‐66‐NH_2_/PdAC. Resin formulations for membrane fabrication were prepared by adding the prepared particle UiO‐66‐NH_2_/PdAC mixtures to CAS8 at varying loadings (10, 20, and 30 wt.%) and stirring for 48 h with intermittent sonication. Immediately before photopolymerization, 1 wt.% BAPO (based on the monomer fraction of the monomer/particle mixtures) was dissolved in the resin formulation.

### Membrane Fabrication

Membranes were fabricated using a Kudo3D Titan 3 digital light processing (DLP) printer to ensure thickness consistency and enable the facile generation of multi‐layer films. Here, the formulated resin was added to a resin vat equipped with a transparent glass window covered with a fluorinated ethylene propylene film, used to ensure facile detachment of the fabricated membrane from the surface at its base. The build stage, equipped with a smooth glass plate to ensure that the fabricated films were flat and untextured, was subsequently positioned at a predetermined distance above the window surface, and the resin was irradiated from below using 385 nm light at an intensity of 8 mW cm^−2^ for 10 min to ensure complete reaction. The build plate was moved up to detach the polymerized film from the vat surface, and the polymerized film was immediately detached from it or, for multi‐layer structures, moved back down to a pre‐determined distance above the vat surface for the fabrication of subsequent layer(s). Before mounting in the separation cell, the fabricated membranes were soaked in toluene overnight.

### Membrane Performance

Fabricated membrane performance was measured using a standard cross‐flow set up (CF047 circular cell assembly, crossflow, 316 SS, Sterlitech Corporation, Auburn, Washington, USA) pressurized with N_2_. The pressure in the system was regulated using an Equilibar GSD4 General Service Back Pressure Regulator. Membranes were affixed to a porous metal support that incorporated an HP4750 PTFE‐encapsulated Viton o‐ring, and this assembly was placed inside the permeation cell. Toluene/MCH mixtures were pressurized on the cell feed side, while the permeate side was maintained at atmospheric pressure. The permeate was collected for permeance calculation. Subsequently, the data were extracted and converted to liters per square meter per hour per bar (L·m^−2^·h^−1^·bar^−1^, LMHbar) values. The permeation (*Q*), rejection (*R*), and separation factor (*SF*) were determined using:

(1) Q=V/A.t.ΔP

(2) $$SF_{a/b}=C_{a,P}/C_{a,R}\times C_{b,R}/C_{b,P}$$

(3) $$R_{a}=1-C_{a,P}/C_{a,R}$$

where *V* is the solvent volume, *A* is the membrane area, *t* is time, Δ*P* is trans‐membrane pressure drop, and *C_P_*, *C_F_*, and *C_R_* are permeate, feed, and retentate concentration, respectively. The compositions of the permeate and retentate were determined using liquid ^1^H NMR analysis, performed on Bruker Avance 400 and 500 MHz NMR spectrometers with the sample held at 25 ± 0.1 °C and a 5 s delay between scans. Samples for NMR spectroscopy were prepared by placing the undiluted toluene/MCH mixtures into 5 mm NMR tubes (i.e., no solvent was used), and the obtained data were processed using MestReNova v14.2.3–29241.

## Conflict of Interest

The authors declare no conflict of interest.

## Author Contributions

A.K. led project administration, and contributed to the conceptual and experimental design, materials synthesis and characterization, execution of experiments, data interpretation, and drafting of the manuscript. F.Z. contributed to project administration, experimental and conceptual design, conducted experiments, performed data analysis, contributed to data interpretation, and co‐drafted the manuscript. M.N.K. led the materials synthesis and characterization. H.M. contributed to materials characterization, data interpretation, and drafting of the manuscript. M.T.S. contributed to materials characterization. D.M.N. contributed to figure design and presentation. B.D.F. contributed to project administration, conceptual and experimental design, and analysis and interpretation of results. M.R.H. contributed to project administration, conceptual and experimental design, data analysis, interpretation of results, and manuscript drafting. T.F.S. led project administration, and contributed to the conceptual and experimental design, data analysis, interpretation of results, and manuscript drafting.

## Supporting information



Supporting Information

## Data Availability

The data that support the findings of this study are available on request from the corresponding author. The data are not publicly available due to privacy or ethical restrictions.
