# Supplementary material for: Photopolymerized Mixed Matrix Membranes for Liquid Organic Hydrogen Carrier Separation
Source: Adv Sci (Weinh). 2025 Aug 28;12(43):e11336. doi: 10.1002/advs.202511336 (PMC12631918; doi:10.1002/advs.202511336)
Supplement: Supplementary file 1 — Supporting Information [file ADVS-12-e11336-s001.docx]

Supporting Information

**Photopolymerized Mixed Matrix Membranes for Liquid Organic Hydrogen Carrier Separation**

*Abdollah Khosravanian^1^, Farnaz Zadehahmadi^2^*, Mohammed Nizam Khan^2^, Hamidreza Mahdavi^1^, Michael T. Scalzo^1^, Declan McNamara^2^, Benny D. Freeman^1,3^, Matthew R. Hill^2^* and Timothy F. Scott^1,2^**

# **Supplementary Methods**

## **1.1 Characterization**

The prepared composite materials (80/20 UiO-66-NH_2_/PdAC) and unfilled and 80/20 UiO-66-NH_2_/PdAC-filled CAS8 membranes were characterized by the following methods.

### 1.1.1 Field-Emission Scanning Electron Microscopy (FE-SEM)

Scanning Electron Microscopy (SEM) was performed using a Thermo Scientific Verios 5 UC FEGSEM. All samples were mounted on aluminium stubs and coated with carbon. The working distance was 4 mm, and images were collected using a 5 kV accelerating voltage. For cross-sectional SEM, the samples were fractured in liquid nitrogen.

### 1.1.2 Fourier Transform Infrared Spectroscopy (FT-IR)

The chemical properties of the composites in both powder form and membrane surface were analyzed using attenuated total reflection Fourier-transform infrared (ATR-FTIR) spectroscopy. A PerkinElmer Spectrum 2 FTIR spectrometer equipped with a UATR diamond/ZnSe crystal was used for the measurements. The spectral range covered 4000–400 cm⁻¹, and the crystal configuration consisted of diamond/ZnSe, with an optional heated liquid analysis capability up to 60 °C.

### 1.1.3 X-ray Photoelectron Spectroscopy (XPS)

X-ray photoelectron spectroscopy (XPS) analysis was performed using the Thermo Nexsa surface analysis system to investigate the elemental composition and chemical states of the UiO-66-NH_2_, 80/20 UiO-66-NH_2_/PdAC, and 50/50 UiO-66-NH_2_/PdAC. The system operates with a monochromatic Al Kα X-ray source (1486.6 eV) under ultra-high vacuum conditions, ensuring high-resolution spectral acquisition. Survey scans were conducted to identify the presence of key elements, while high-resolution spectra of relevant core-level peaks provided insights into their chemical bonding states. The deconvolution of these spectra enabled the identification of different oxidation states and possible interactions between N, H and Pd elements in the solid mixtures.

### 1.1.4 Proton Nuclear Magnetic Resonance (1H-NMR)

To evaluate the separation performance of the membranes, ^1^H-NMR spectroscopy was employed to analyze the feed, permeate, and retentate samples. The separation factor of the membranes was determined by comparing the ratio of the integrals between the toluene CH_3_ peak and the methylcyclohexane (MCH) peaks in the samples from the toluene-methylcyclohexane separation process. NMR spectra were acquired using an Av400 NMR or an Av500P NMR spectrometer, with a 5-second delay between scans, and without the use of any solvents.

### 1.1.5 Powder X-ray Diffraction (PXRD)

PXRD was conducted to measure crystallinity of the samples using a Bruker D8 Advance A25 Diffractometer. The scans used CuKα radiation (40 kV, 40 mA) with a step size of 0.02°, 0.10 s/step, and scanned from 5–80°. The sample was spun at 15 rpm and data were collected using a Lynx Eye XE-T detector.

### 1.1.6 Zeta Potential Measurements

An Anton Paar SurPass 3 streaming zeta potential instrument was used for the surface charge analysis of UiO-66-NH_2_ and PdAC powders (the average of three repeats on each sample).

### 1.1.7 Atomic Force Microscopy (AFM)

AFM-IR measurements were conducted using a NanoIR2 system (Anasys Instruments Inc., Santa Barbara, USA). The system utilized an optical parametric oscillator (OPO) laser as the IR source, generating 10 ns pulses at a repetition rate of 1 kHz. A silicon cantilever (AppNano, Mountain View, CA, USA) with a nominal radius of 10 nm and a spring constant of 0.5 N/m was employed for the measurements. To maintain controlled humidity conditions, the system was purged with nitrogen.

### 1.1.8 Gas and vapour uptake tests

Gas uptake of the prepared 80/20 UiO-66-NH_2_/PdAC mixtures were tested on a Micromeritics ASAP 2460 instrument to investigate the pore properties. The N_2_ adsorption experiments were conducted at 77 K.

Vapor adsorption isotherms were conducted using a Micromeritics 3Flex. Isotherms were obtained in a room-temperature water bath, and each run was terminated before the vapor pressure of each solvent.

### 1.1.9 Rheometry

#### Viscosity measurement

The viscosities of the prepared composites with 10, 20, and 30 wt. % filler loadings were measured at different temperatures (20, 35 and 50 °C) and shear rates (0.1-1000 1/s) using Anton Paar Physica rheometer equipped with a parallel plate measuring system. The composite was placed between a 25 mm measuring system on top and quartz on the bottom with the gap of 0.3 mm. The pre-shear time was set to 1 min.

#### Chemorheology

The storage modulus data were obtained using an Anton Paar Physica rheometer equipped with a parallel plate photocuring system. The composite was positioned between a 12 mm measuring system on top and quartz on the bottom, maintaining a 0.05 mm gap. UV light, with an intensity of 8 mW/cm², was emitted from an Omnicure 2000 lamp equipped with a 400–500 nm bandpass filter. UV light intensity was calibrated using an International Light IL1400A radiometer. The tests were conducted under 0.1% strain and 1 Hz frequency conditions. The samples were left in the dark for 15 seconds before opening the shutter for irradiation.

### 1.1.10 Dynamic mechanical analysis (DMA)

Membranes with dimensions of 20 mm × 3 mm × 0.25 mm were analyzed in a TA Instruments DMA Q850 dynamic mechanical analyzer (DMA), which was equipped with a liquid nitrogen cooling accessory for storage modulus temperature dependence and glass transition temperature (*T*_g_) analysis. The temperature scanning ranged from -90 °C to 100 °C at a 2 °C/min rate, with a 0.1% strain and 1 Hz frequency.

### 1.1.11 Fourier Transform Infrared Spectroscopy (FTIR) Kinetics Study

The reaction formulations were laminated between polished NaCl plates and characterized using a Thermo Scientific Nicolet 6700 FTIR. Samples were irradiated after 10 seconds with a 365 nm collimated LED source (Thorlabs M365LP1-C1) controlled by an adjustable LED driver (Thorlabs LEDD1B) with an intensity of 20 mW/cm^2^. Irradiation intensities were measured using an International Light IL1400A radiometer with a GaAsP detector (model SEL005), a 10× attenuation neutral density filter (model QNDS1), and a quartz diffuser (model W). Conversions were monitored in real-time in the mid-IR region (650 to 4000 cm^-1^) at a rate of 2 scans per second and a resolution of 2 cm^-1^.

# **Supplementary Data and Figures**


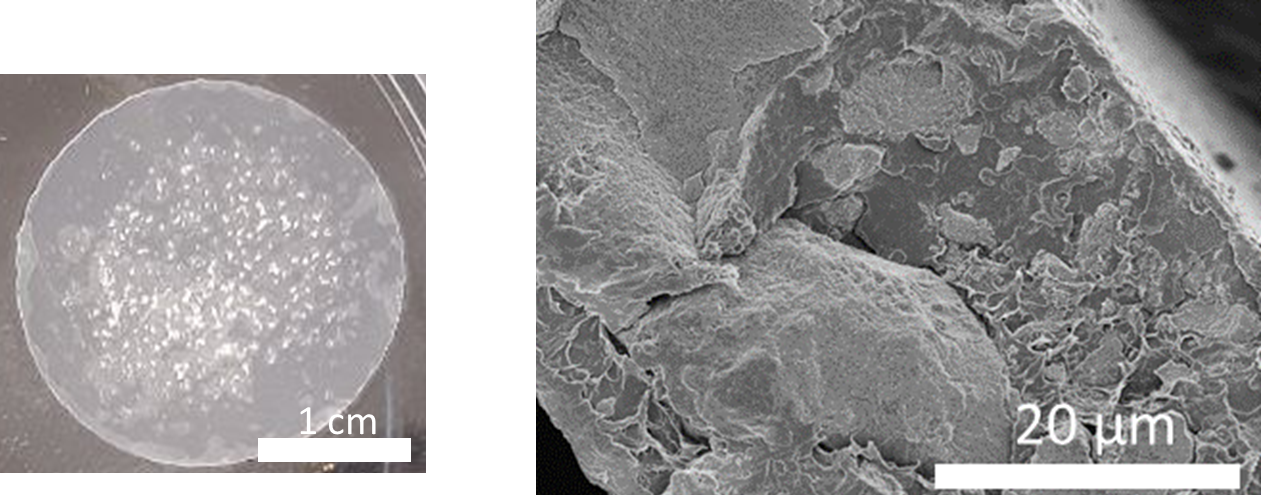


a)

**
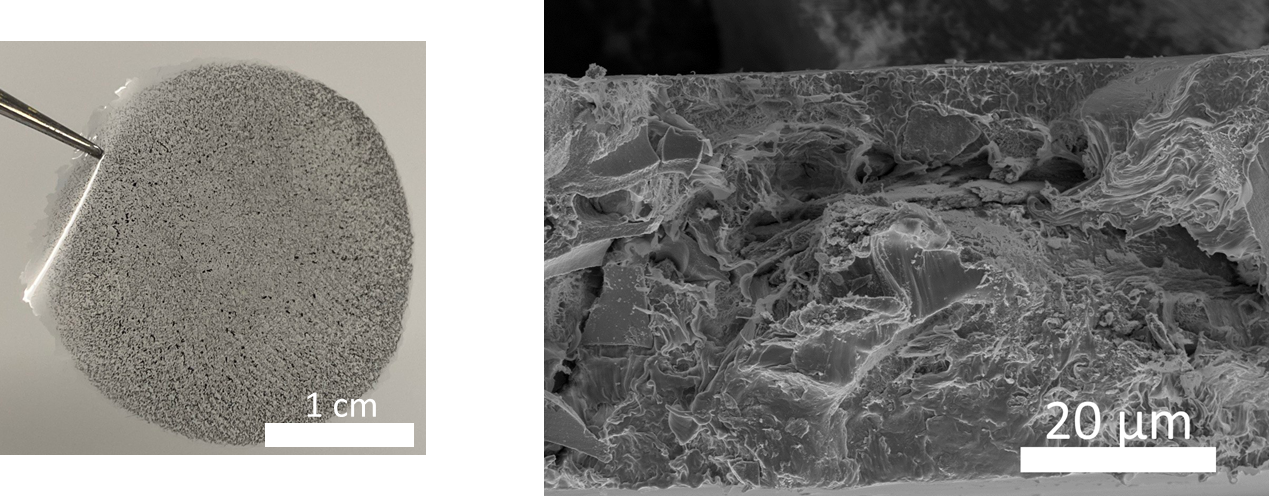
**

b)


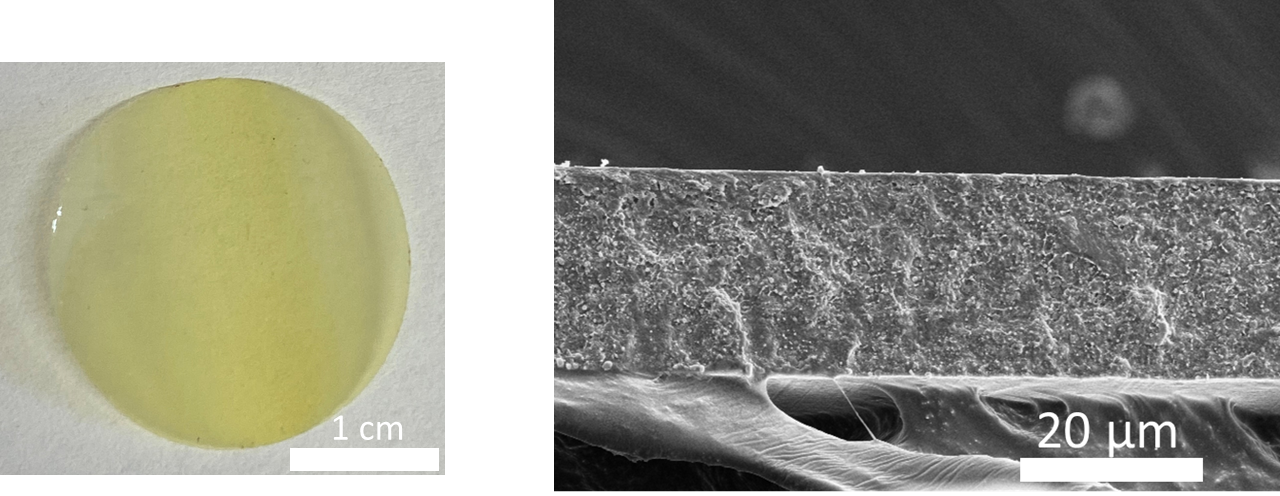


c)

Figure S1.Photographs and SEM images of MOF- and PdAC-filled, photopolymerized CAS8 composite films. a) 20 wt. % UiO-66 in CAS8, b) 4 wt. % PdAC in CAS8, and c) 20 wt. % UiO-66-NH_2_ in CAS8.


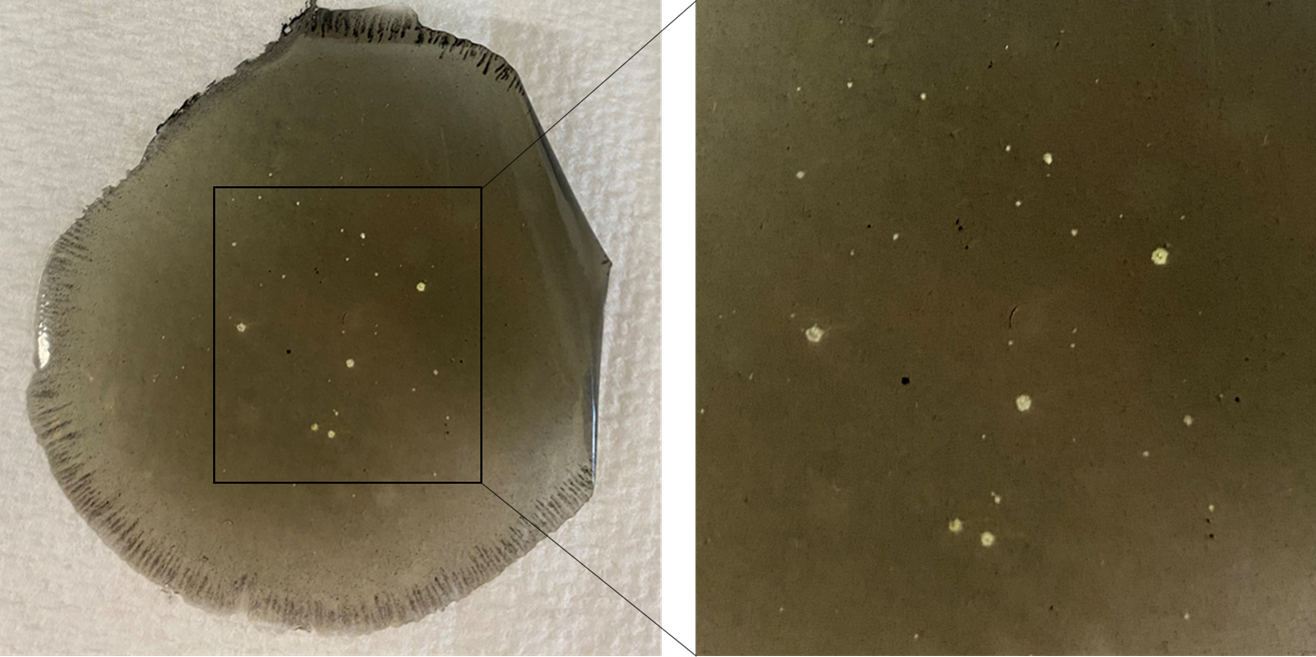


Figure S2. Photograph of photopolymerized composite film composed of 30 wt. % 80/20 UiO-66-NH_2_/PdAC, (prepared with less than 5 hours of solid mixing) in CAS8.


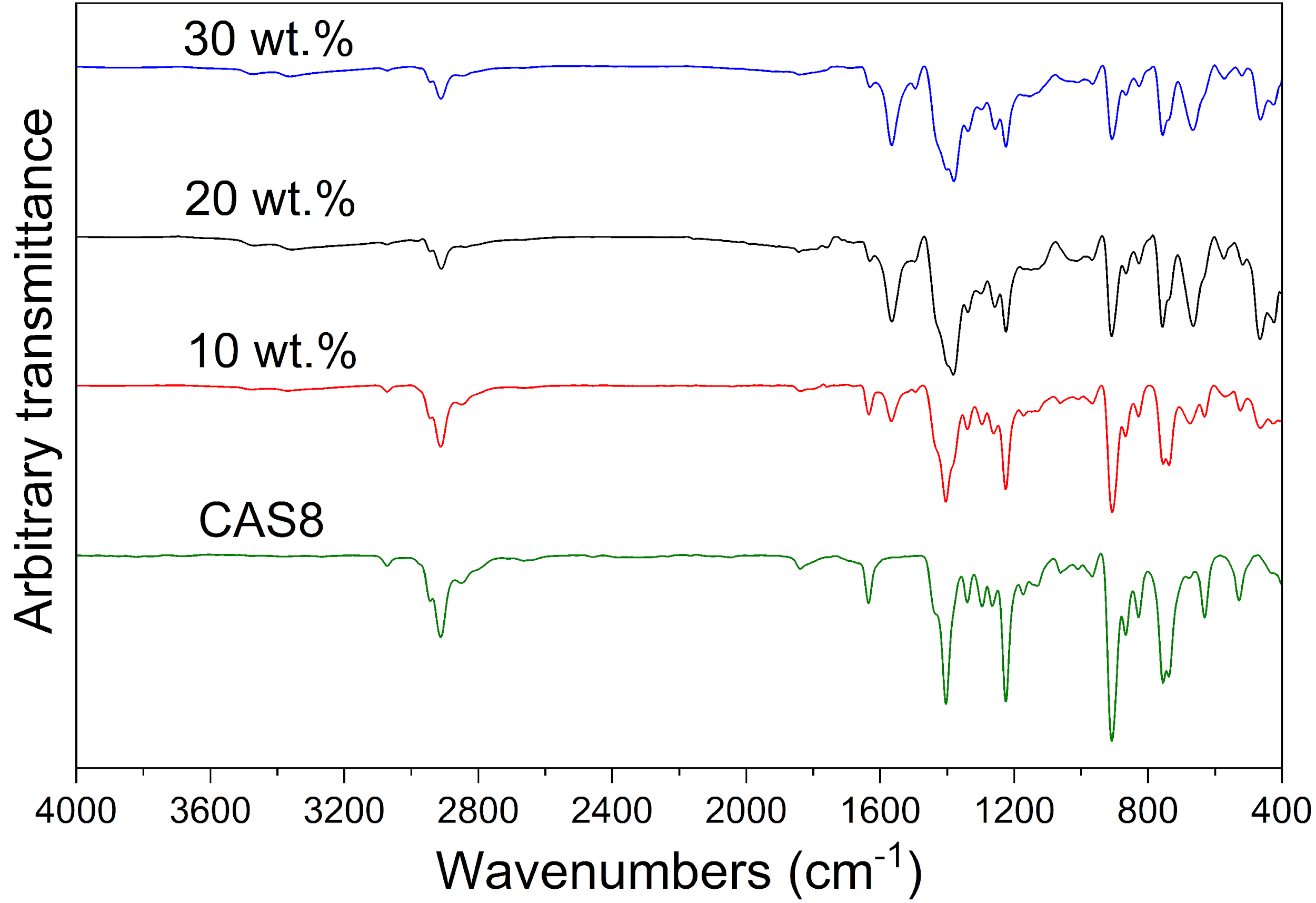


a)


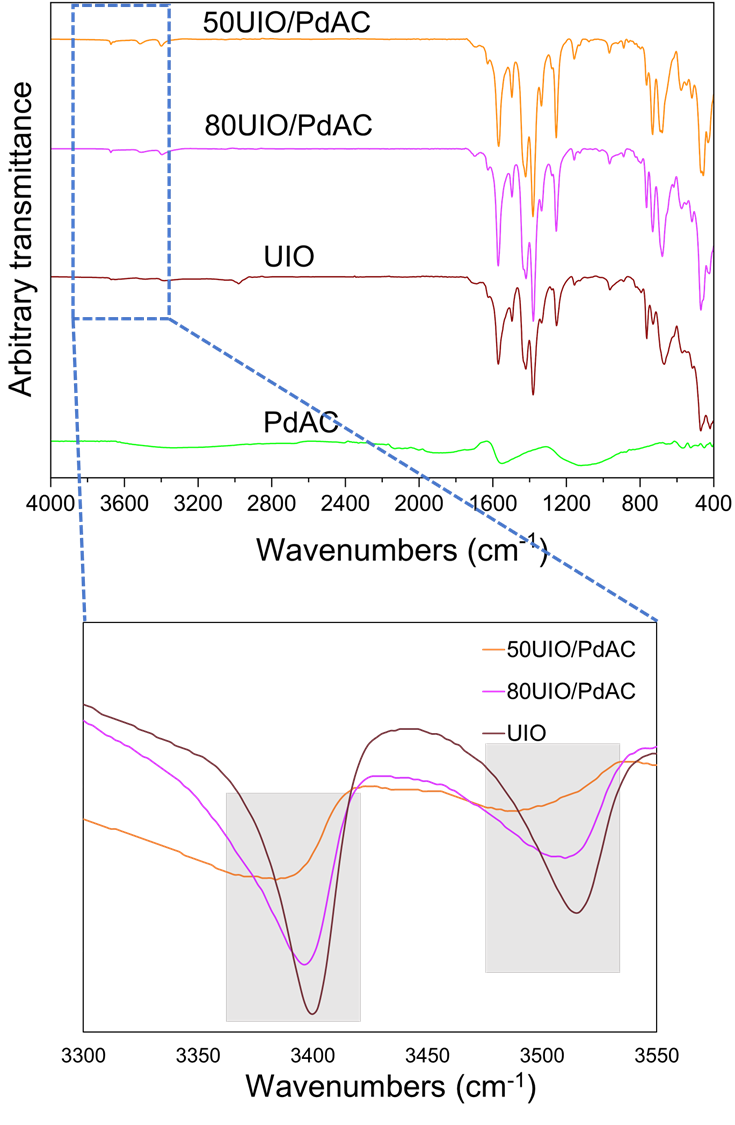


b) & c)

Figure S3. Fourier transform infrared (FT-IR) spectra of particles, particle mixtures, and polymerized films. a) Transmittance spectra of 50 μm thick photopolymerized films of unfilled CAS8 and 10, 20 and 30 wt. % 80/20 UiO-66-NH_2_/PdAC in CAS8. b) Diffuse reflectance spectra of PdAC, UiO-66-NH_2_, 80/20 UiO-66-NH_2_/PdAC, and 50/50 UiO-66-NH_2_/PdAC powders. c) Magnified region of particle spectra.


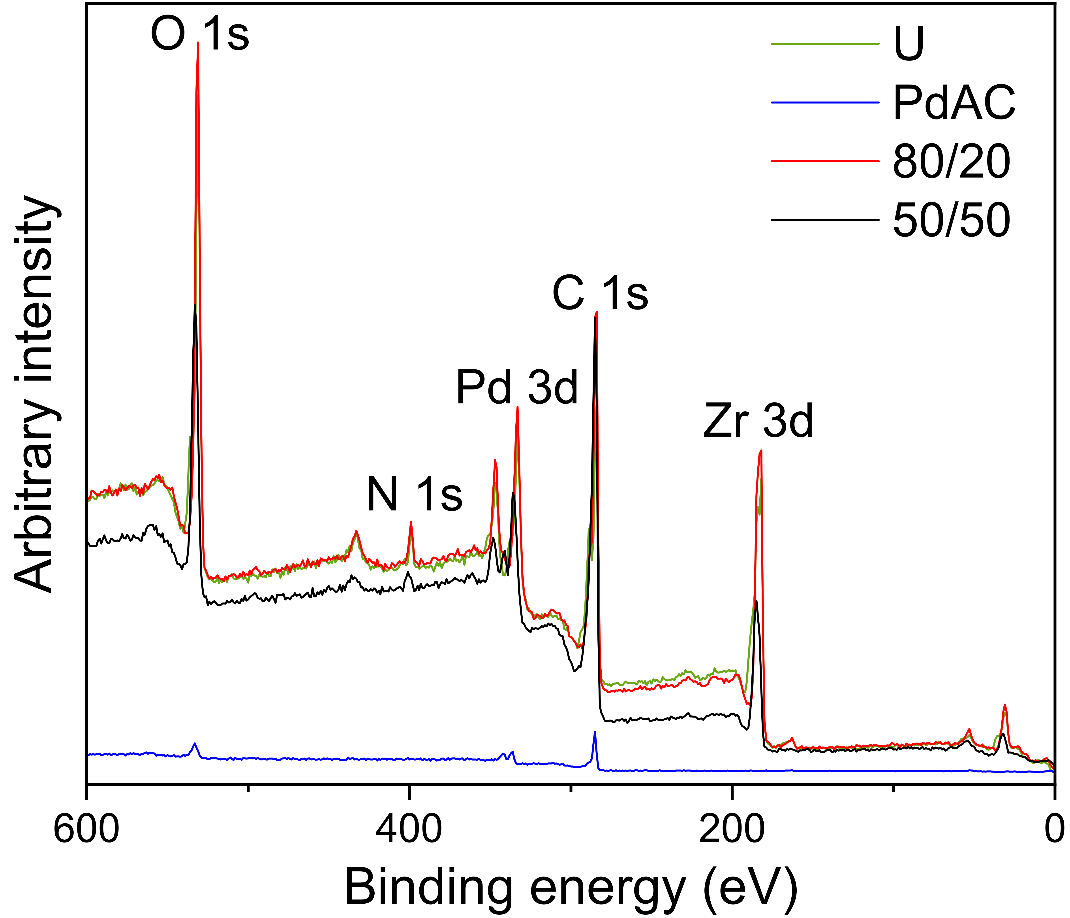


a)
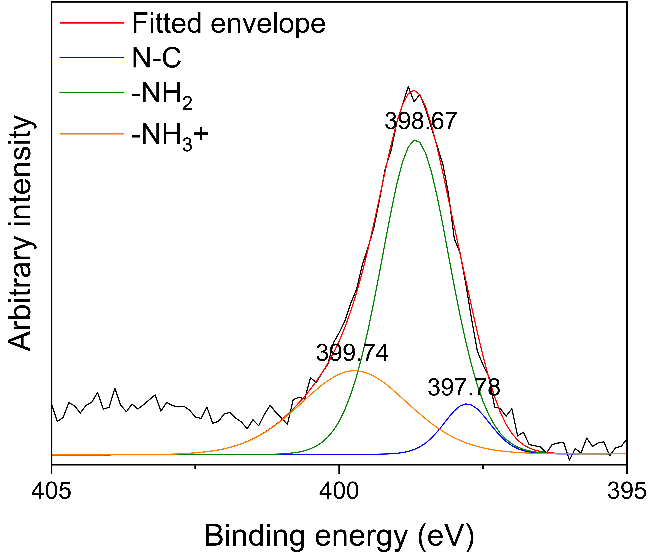
b)
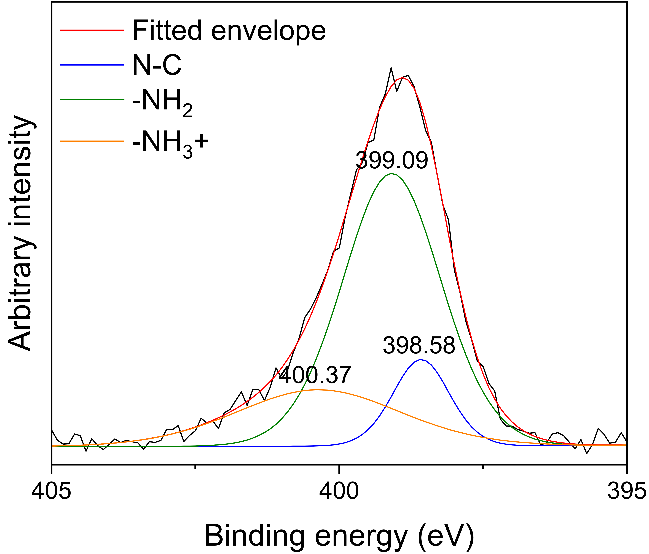


c)
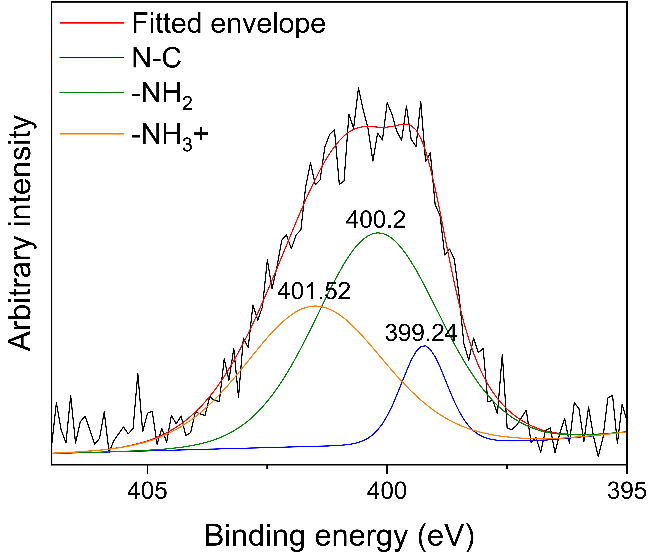
d)
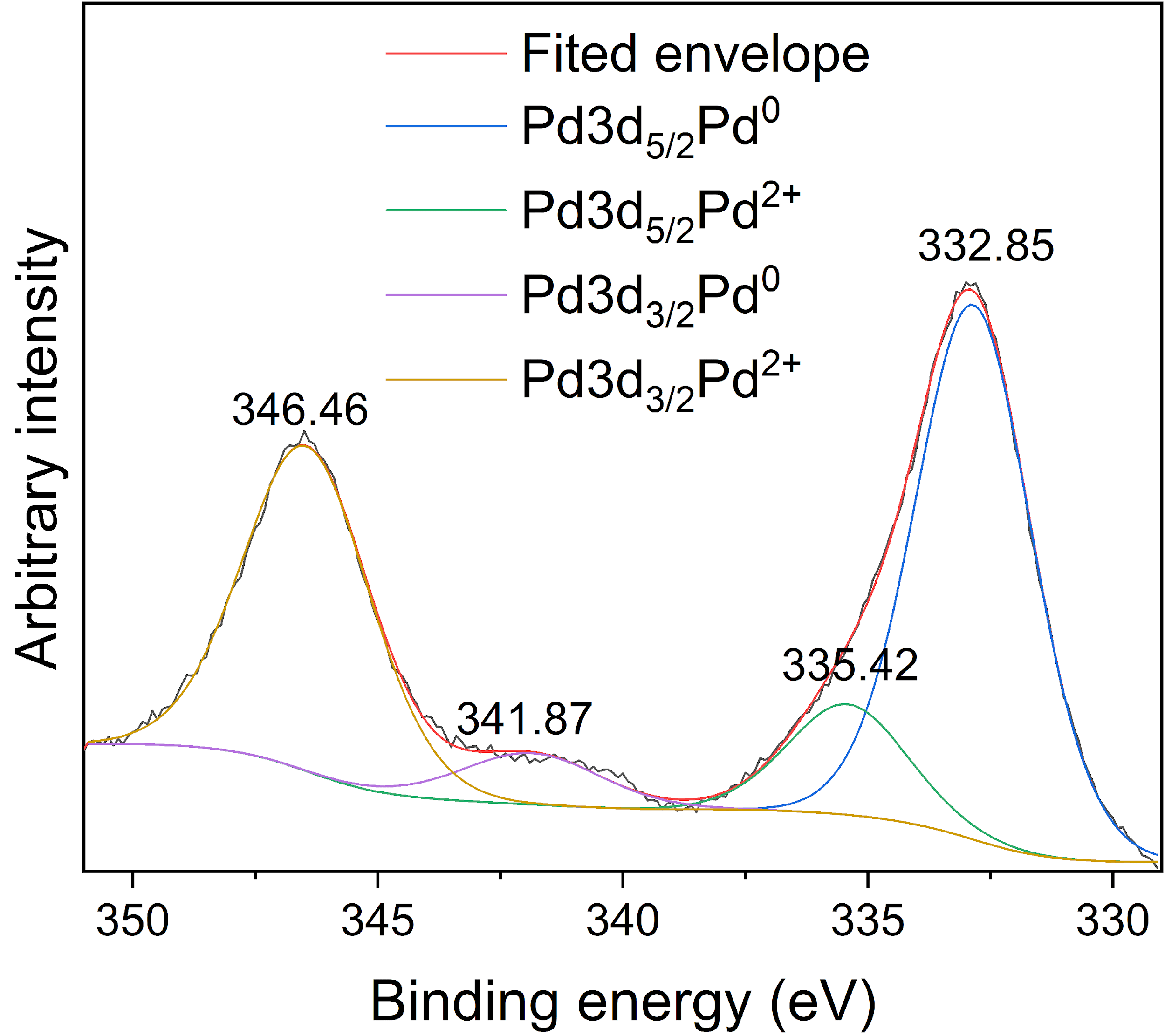


Figure S4. XPS analysis with N 1s deconvolution (magnified insets) for the solid mixtures, confirming chemical interactions between UiO-66-NH_2_ and PdAC in a) UiO-66-NH_2_, b) 80/20 UiO-66-NH_2_/PdAC, and c) 50/50 UiO-66-NH_2_/PdAC. The electronic structure of Pd shown in d) indicates the presence of both metallic and oxidized Pd species.


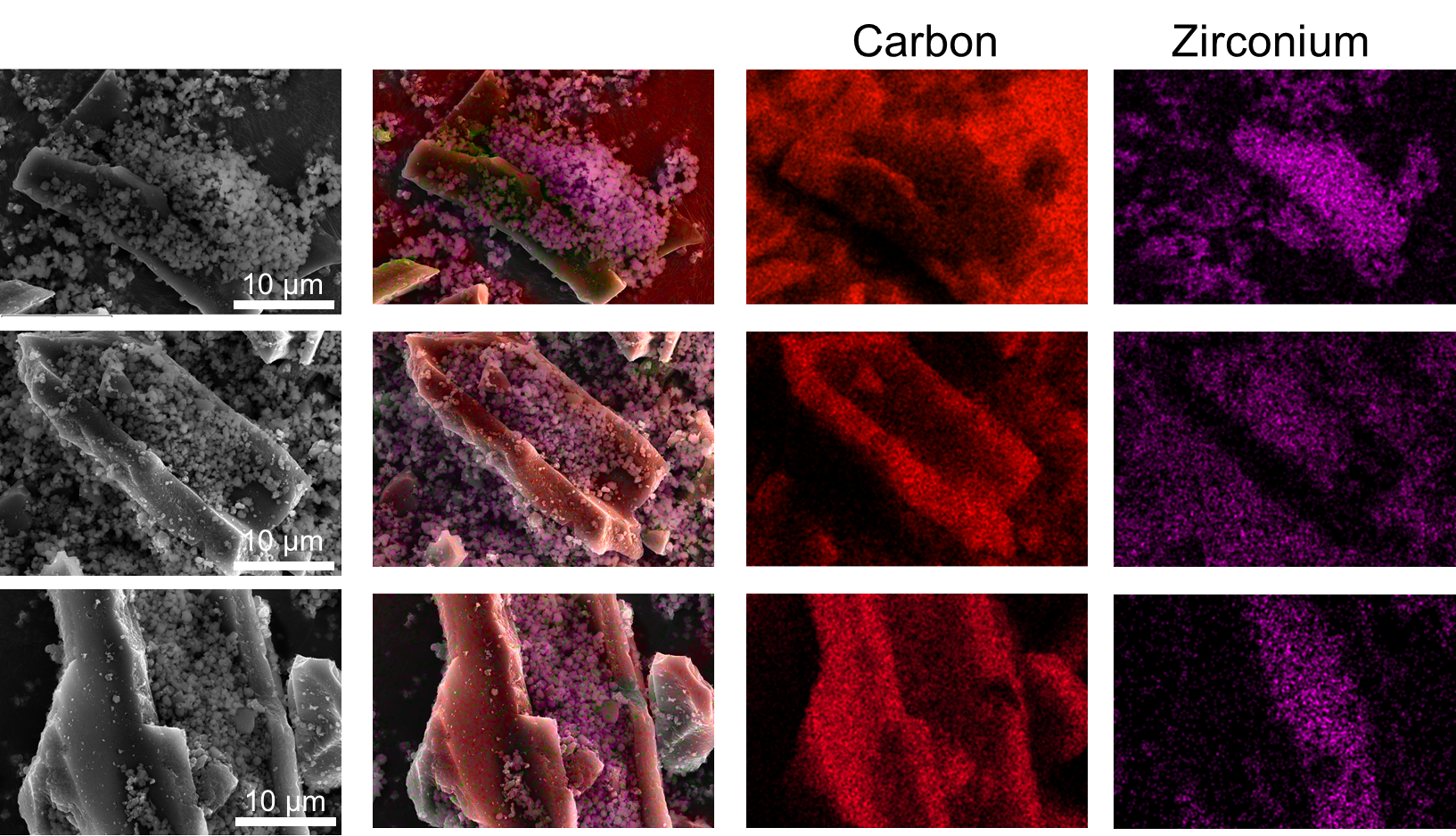


Figure S5. EDX mapping of 80/20 UiO-66-NH_2_/PdAC particle mixture


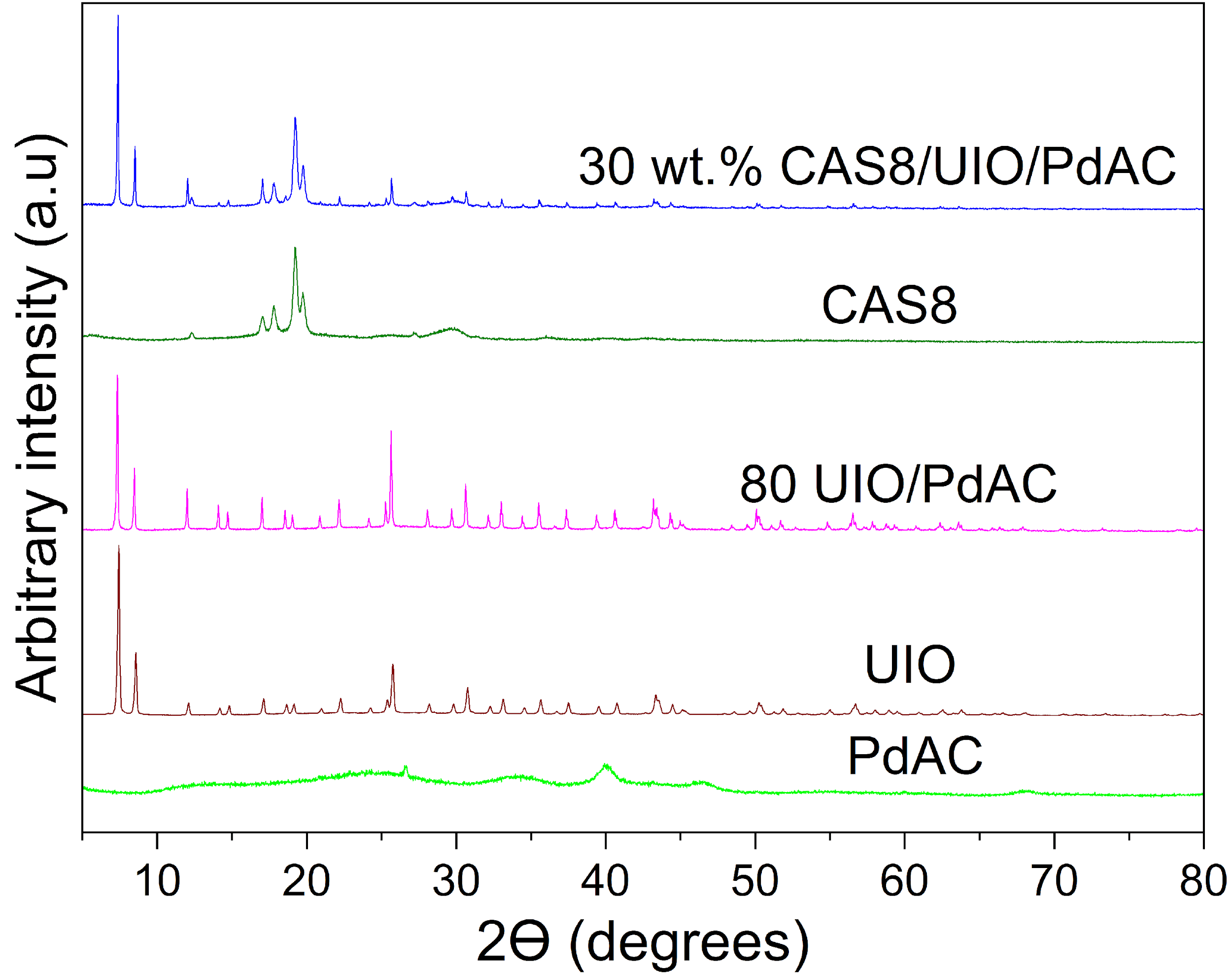


Figure S6. XRD patterns of PdAC, UiO-66-NH_2_, and 80/20 UiO-66-NH_2_/PdAC composites powders, unfilled CAS8 membrane and 30 wt. % of 80/20 UiO-66-NH_2_/PdAC in CAS8 membrane.


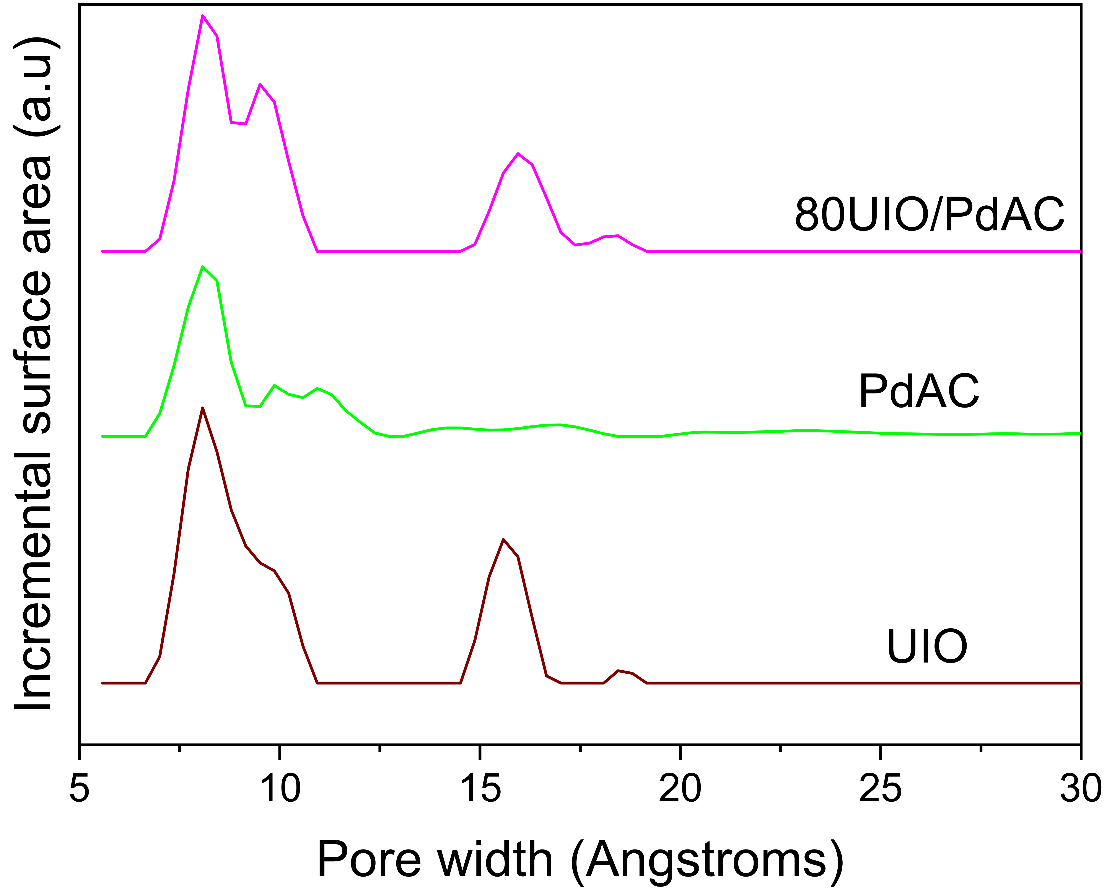


Figure S7. Pore size distribution of UiO-66-NH_2_, PdAC, and UiO-66-NH_2_/PdAC, determined by nitrogen adsorption–desorption isotherms using the BET method.


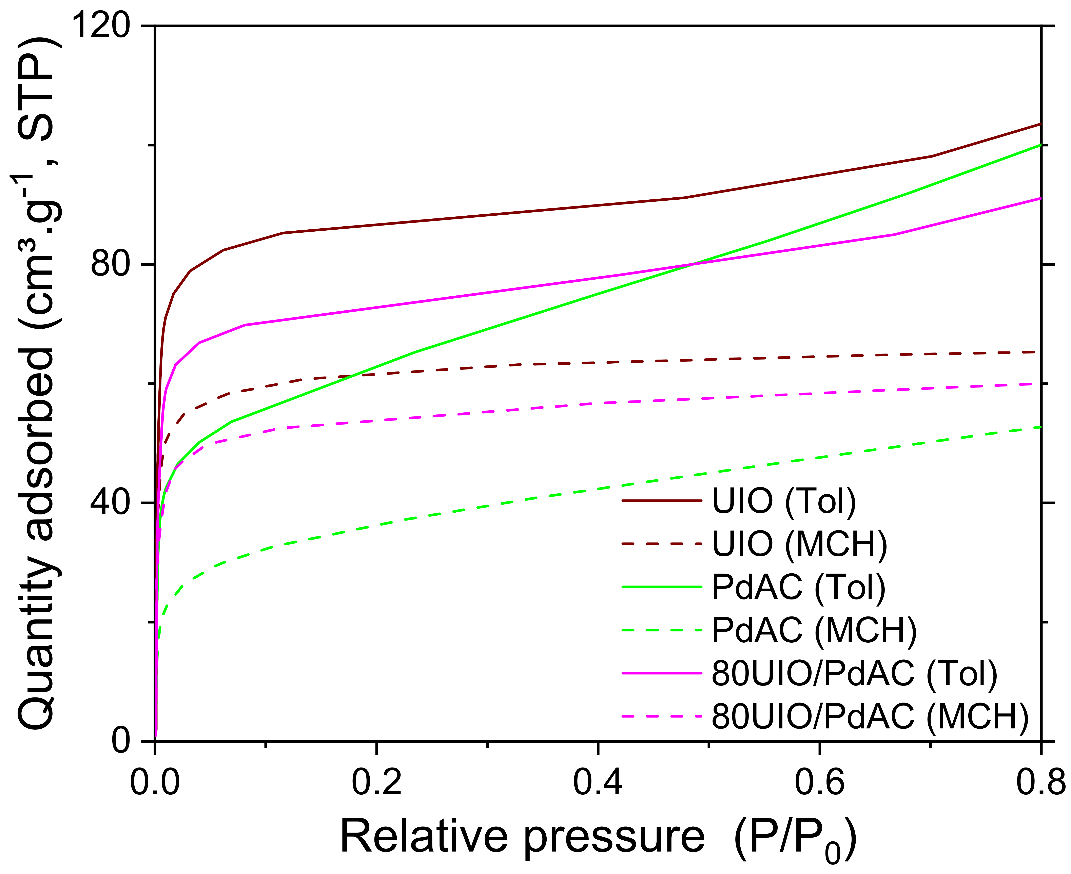


Figure S8. Toluene and methylcyclohexane vapour adsorption at 298 K by UiO-66-NH_2_, PdAC, and 80/20 UiO-66-NH_2_/PdAC.


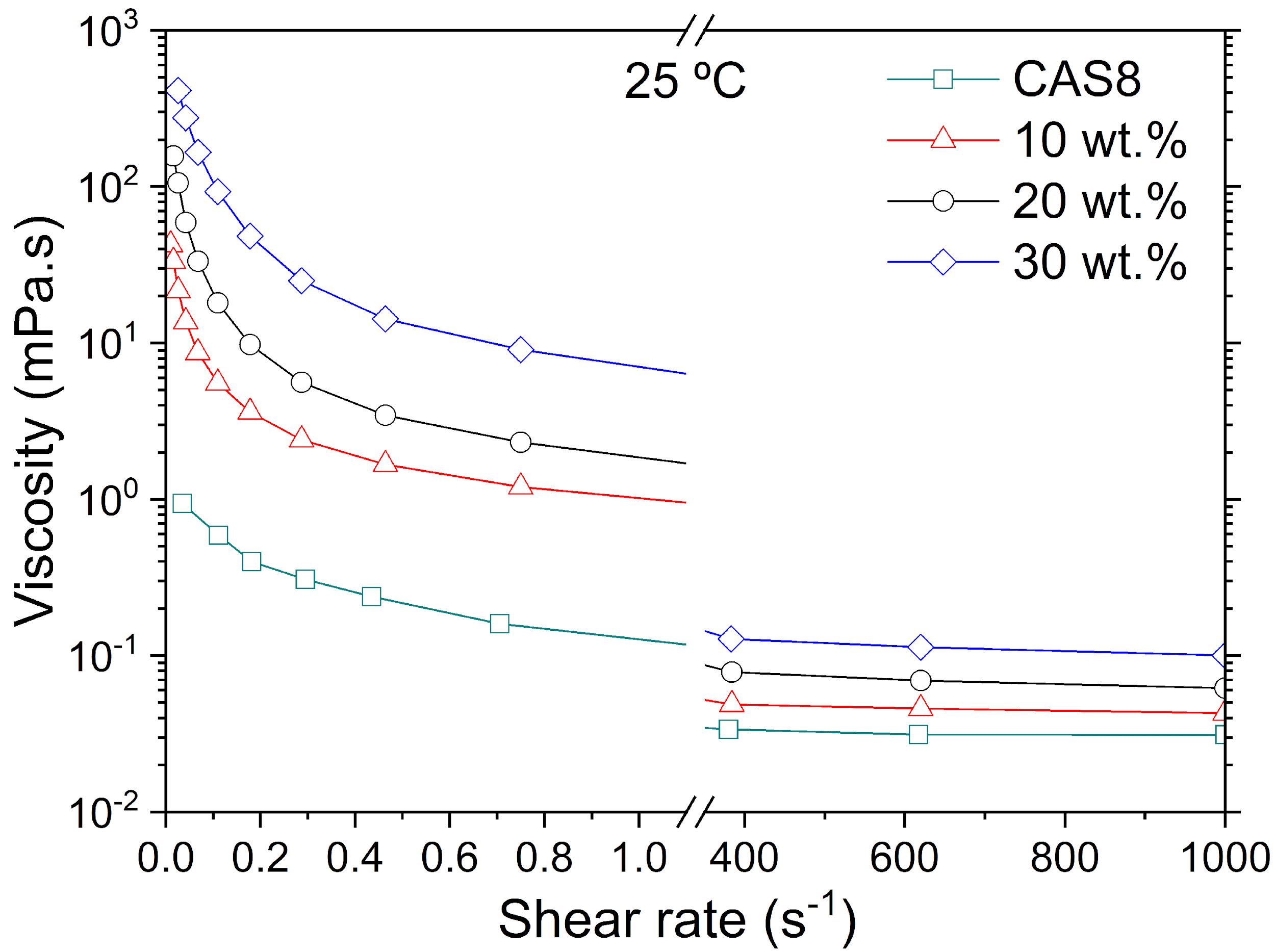


Figure S9. Viscosity vs shear rate for unfilled CAS8 and 10, 20, and 30 wt. % 80/20 UiO-66-NH_2_/PdAC in CAS8 at 25 °C.


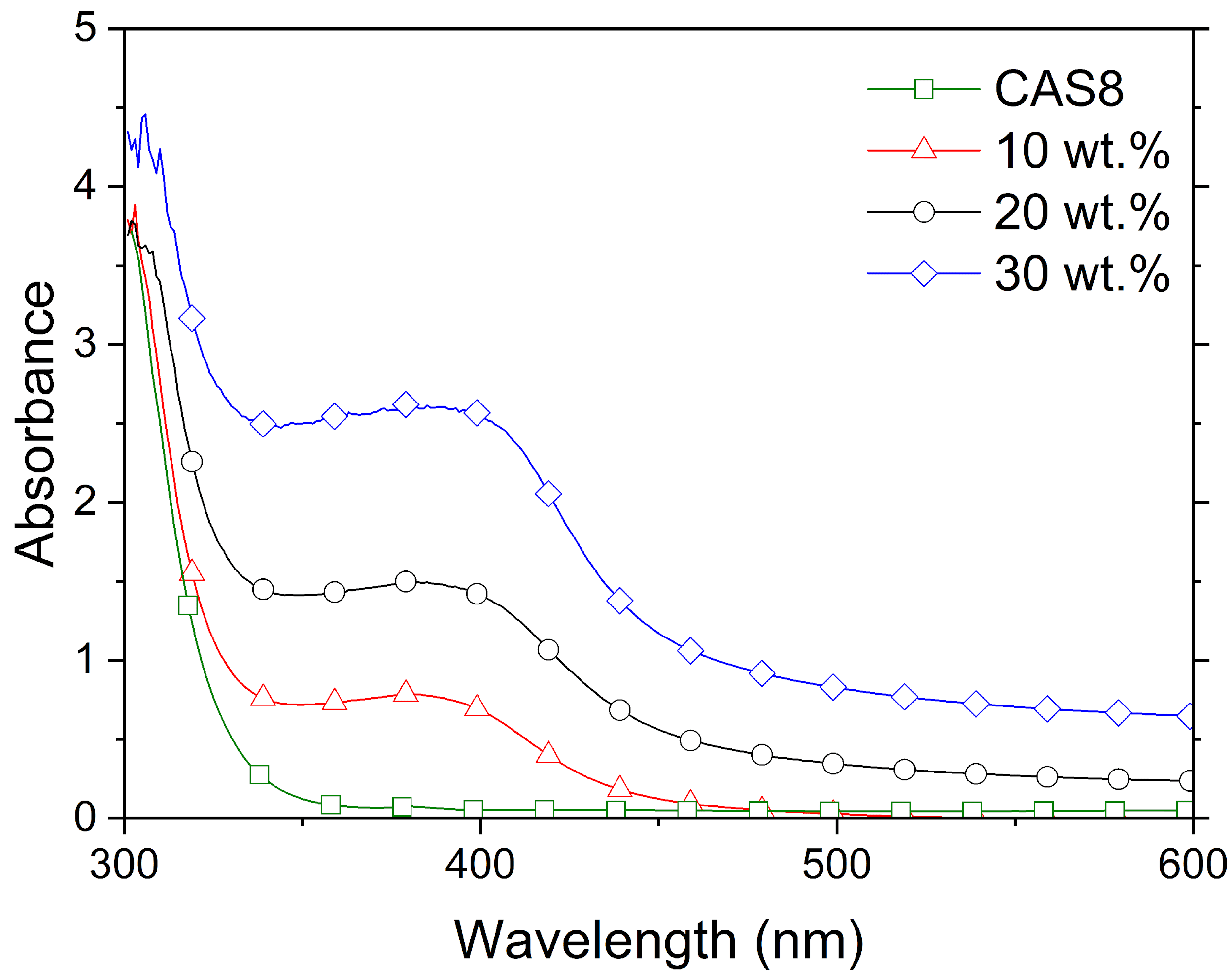


Figure S10. Ultraviolet-visible (UV-vis) absorbance spectra of unfilled and UiO-66-NH_2_/PdAC-filled CAS8 monomer formulations using a pathlength of 15 um.


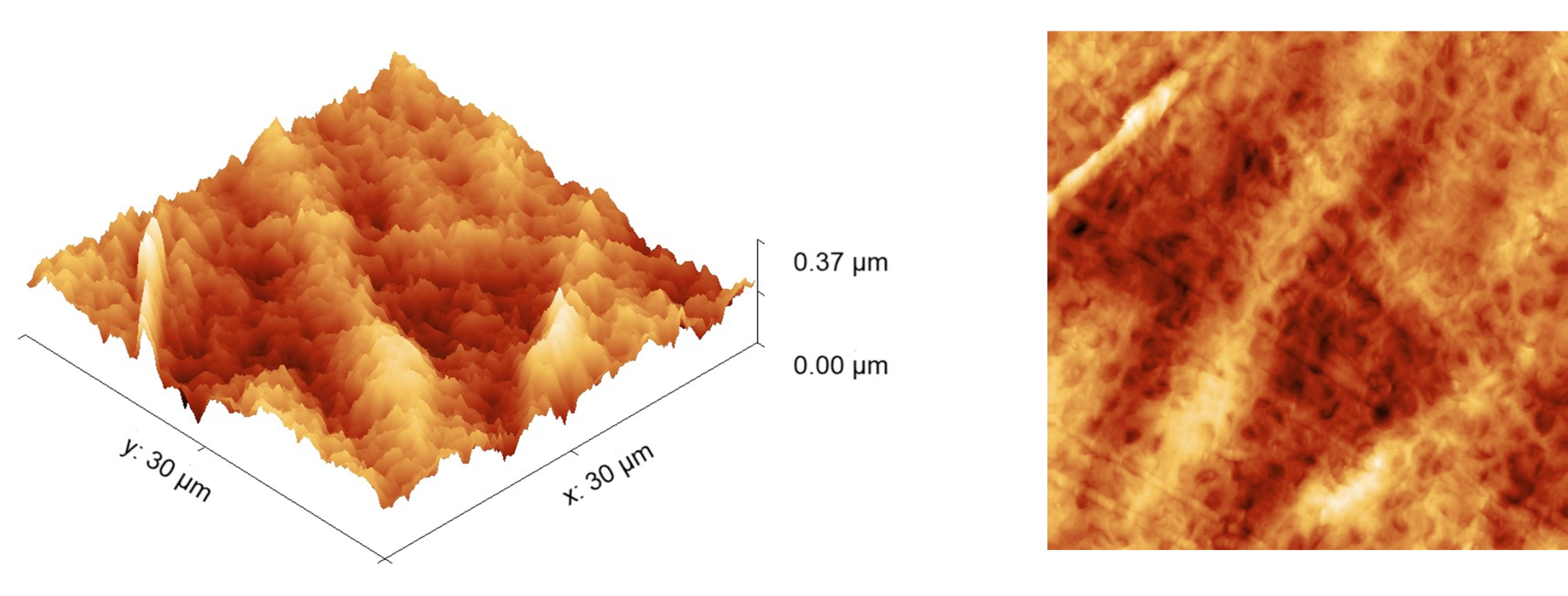


a)


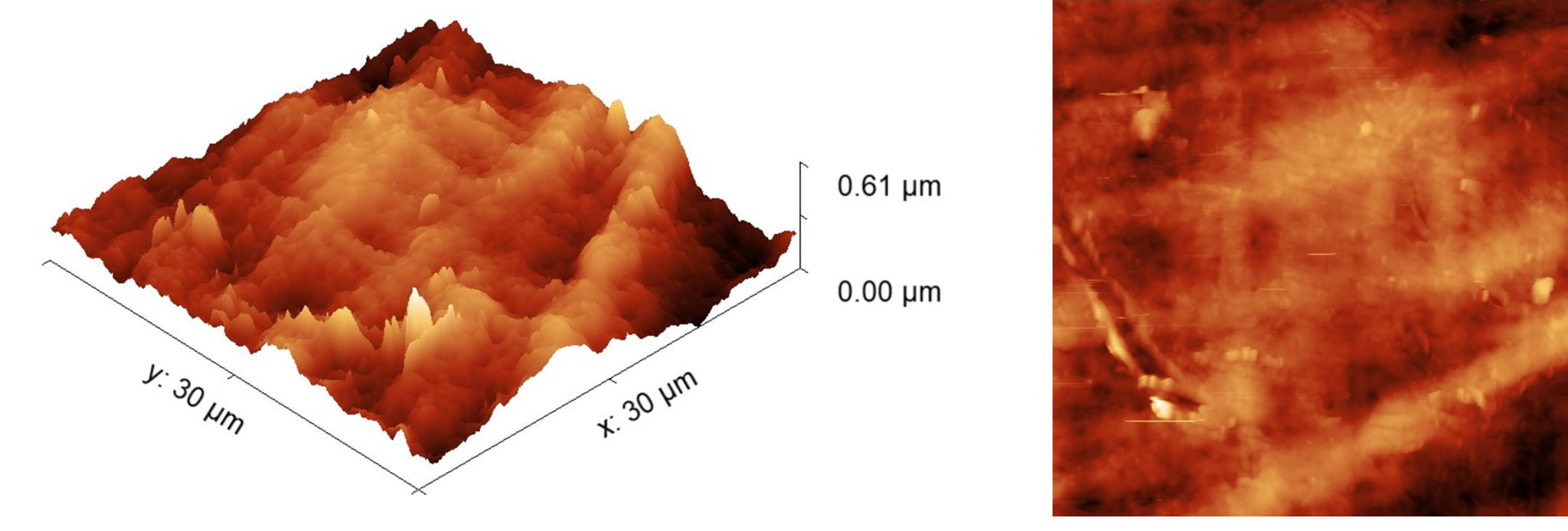


b)


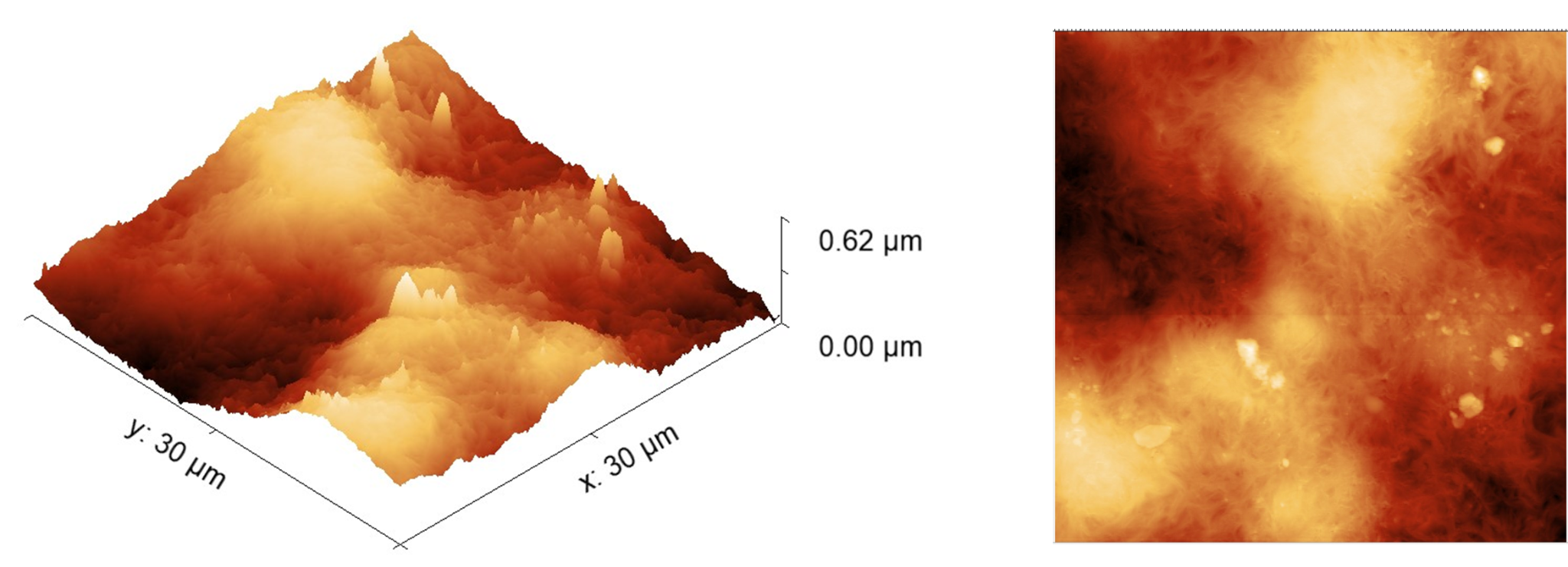


c)


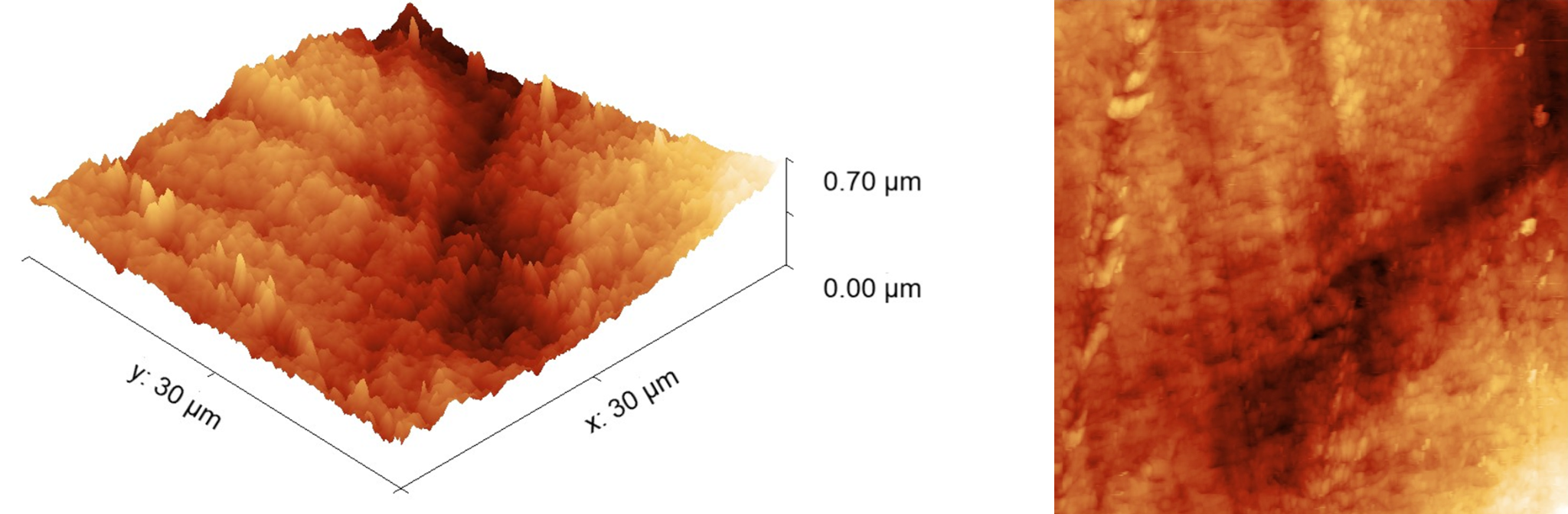


d)

Figure S11. AFM images of the fabricated membranes, a) unfilled CAS8, b) 10, c) 20, and d) 30 wt. % of 80/20 UiO-66-NH_2_/PdAC loaded in CAS8.


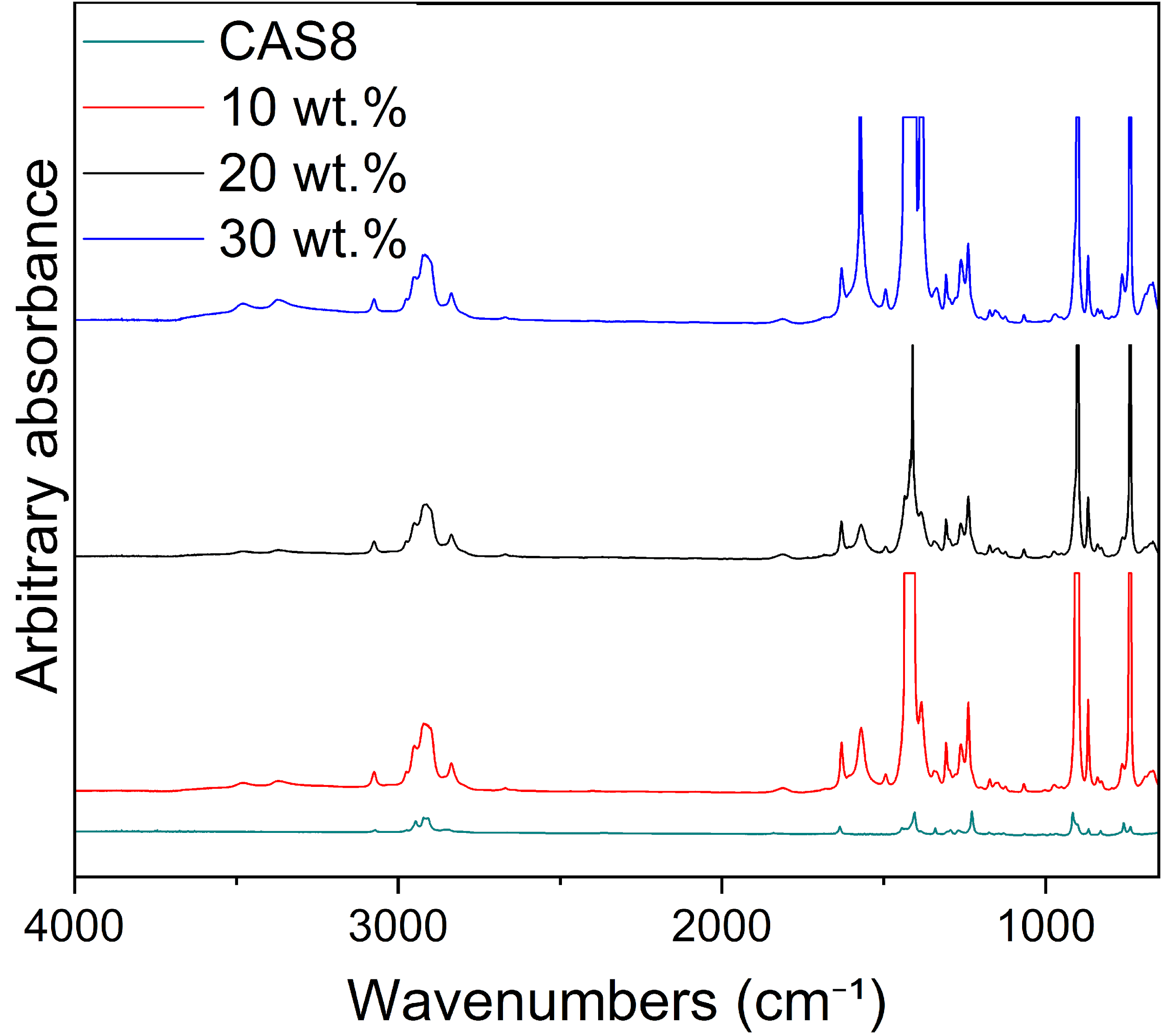


a)

**
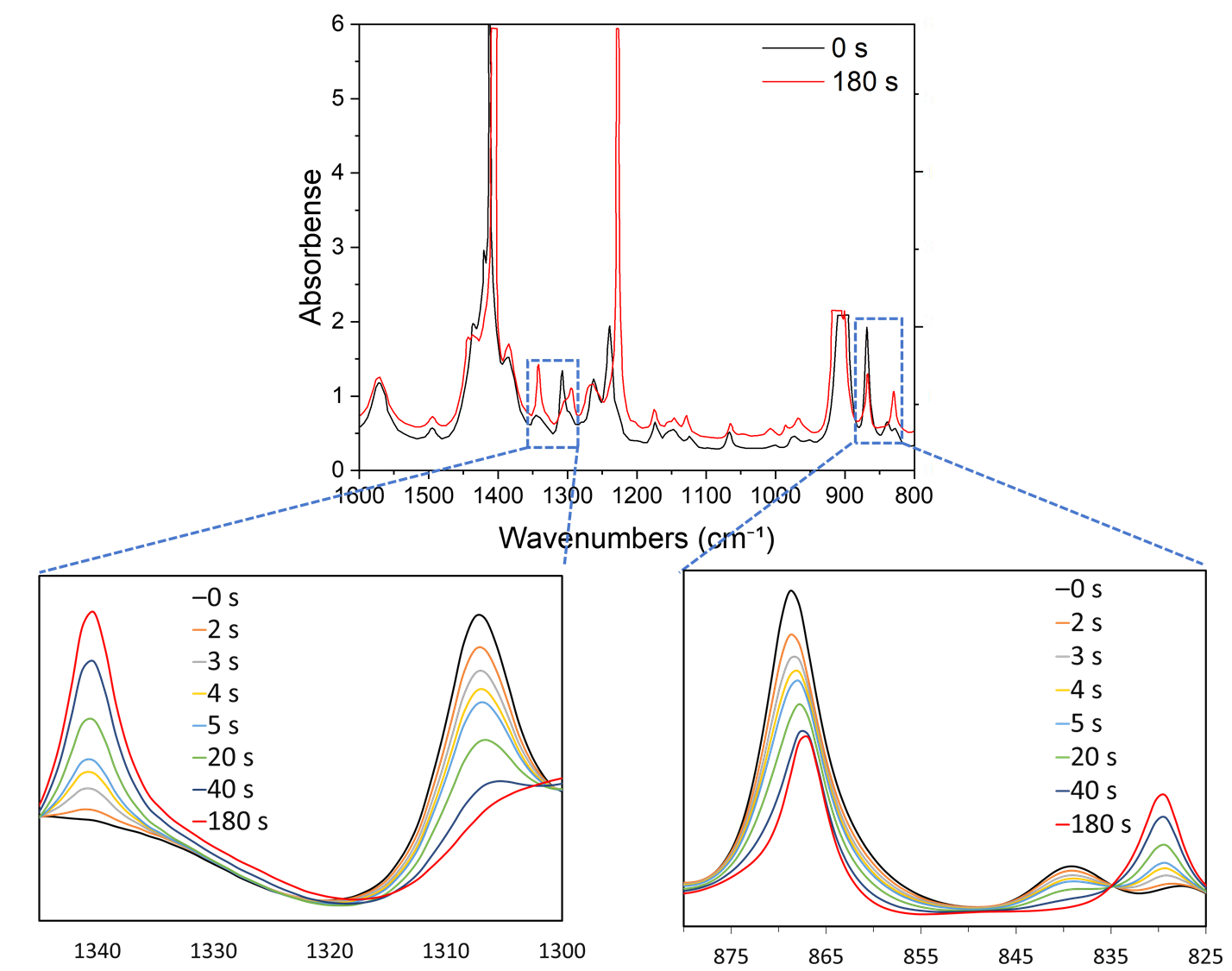
**

b)

Figure S12. FTIR spectra of (a) unfilled CAS8 and CAS8 with 10-30 wt. % 80/20 UiO-66-NH_2_/PdAC and (b) FTIR spectra of CAS8 with 20 wt. % 80/20 UiO-66-NH_2_/PdAC prior to light irradiation (black) and after 3 mins of light irradiation with 405 nm light at 8 mW/cm^2^ (red) in conjunction w

**
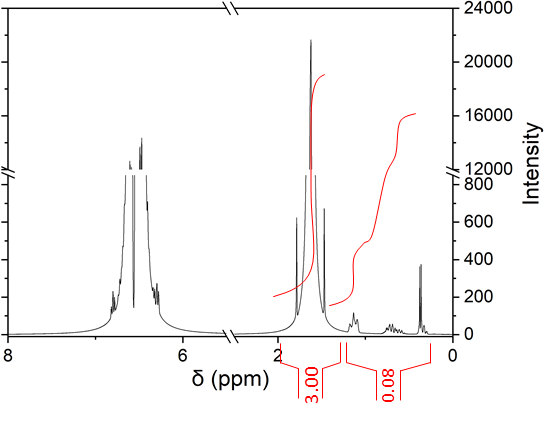
**

a)

**
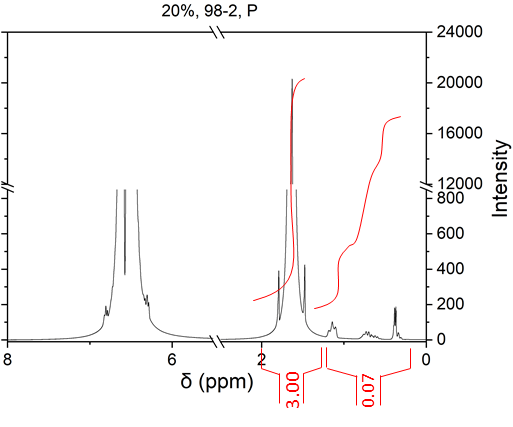
**

b)

**
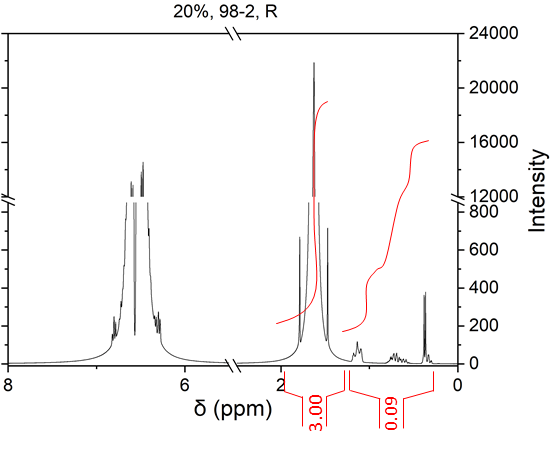
**

c)

Figure S13. 1H-NMR spectrum of the feed, permeate and retentate with 20 wt. % 80/20 UiO-66-NH_2_/PdAC in CAS8 membrane, Tol/MCH ~ 98:2 molar ratio. a) feed, b) permeate and c) retentate. It should be noted that the resonance peak at ~1.6 ppm corresponds to the protons in Tol and the resonance peaks at ~0.4-1.2 ppm correspond to the protons in MCH.

**
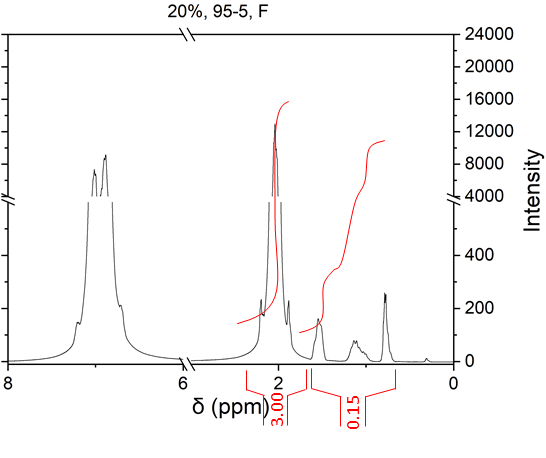
**

a)

**
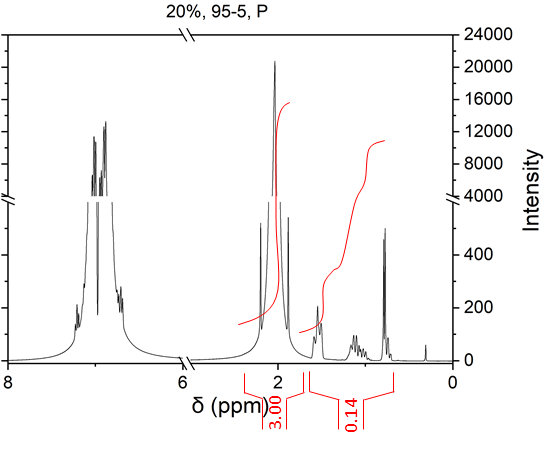
**

b)

**
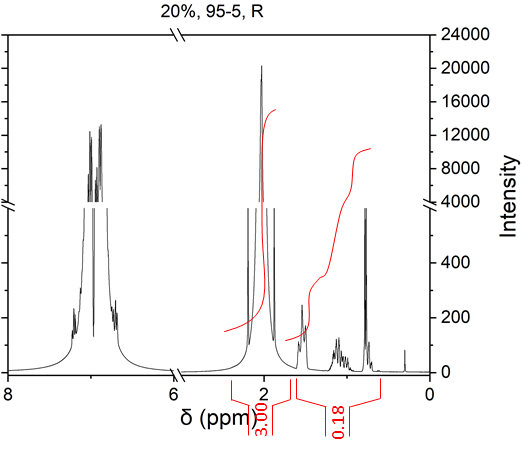
**

c)

Figure S14. 1H-NMR spectrum of the feed, permeate and retentate with 20 wt. % 80/20 UiO-66-NH_2_/PdAC in CAS8 membrane, Tol/MCH ~ 95:5 molar ratio. a) feed, b) permeate and c) retentate. It should be noted that the resonance peak at ~2 ppm corresponds to the hydrogens in Tol and the resonance peaks at ~0.8-1.6 ppm correspond to the hydrogens in MCH.

**
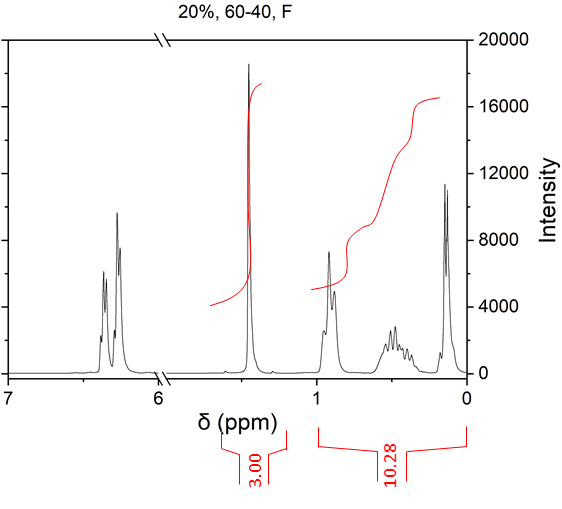
**

a)

**
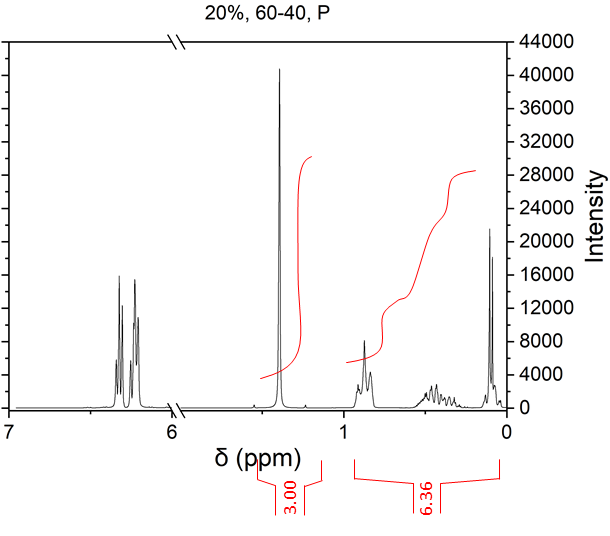
**

b)

**
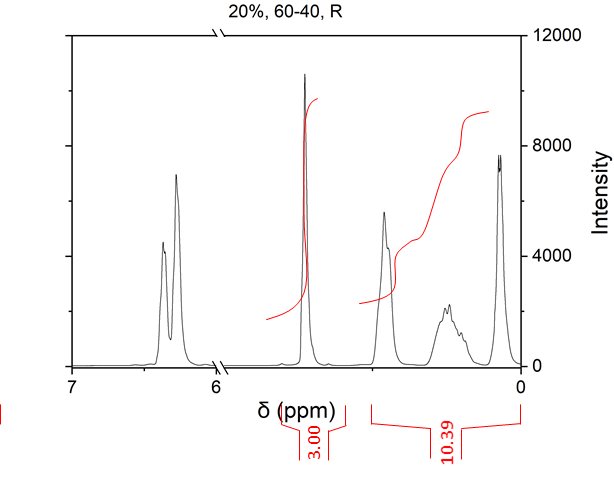
**

c)

Figure S15. 1H-NMR spectrum of the feed, permeate and retentate with 20 wt. % 80/20 UiO-66-NH_2_/PdAC in CAS8 membrane, Tol/MCH ~ 60/40 molar ratio. a) feed, b) permeate and c) retentate. It should be noted that the resonance peak at ~1.5 ppm corresponds to the hydrogens in Tol and the resonance peaks at ~0-1.2 ppm correspond to the hydrogens in MCH.

**
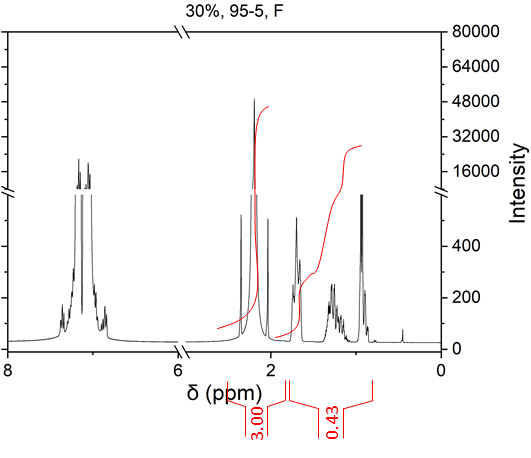
**

a)

**
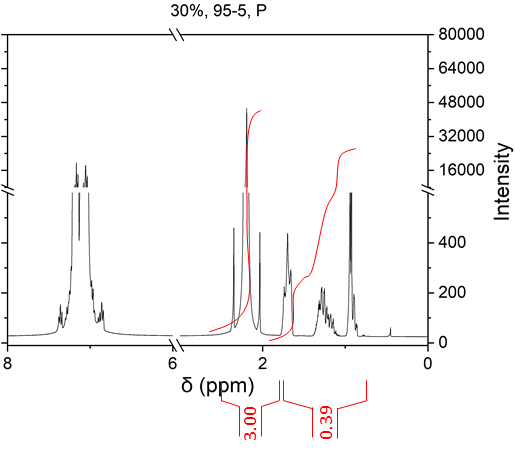
**

b)

**
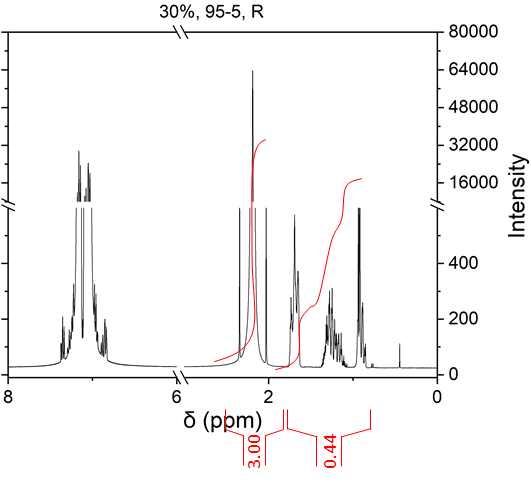
**

c)

Figure S16. 1H-NMR spectrum of the feed, permeate and retentate with 30 wt. % 80/20 UiO-66-NH_2_/PdAC in CAS8 membrane, Tol/MCH ~ 95/5 molar ratio. a) feed, b) permeate and c) retentate. It should be noted that the resonance peak at ~2.2 ppm corresponds to the hydrogens in Tol and the resonance peaks at ~0.7-1.7 ppm correspond to the hydrogens in MCH.

**
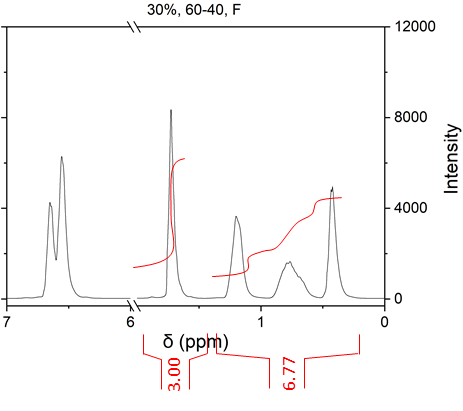
**

a)

**
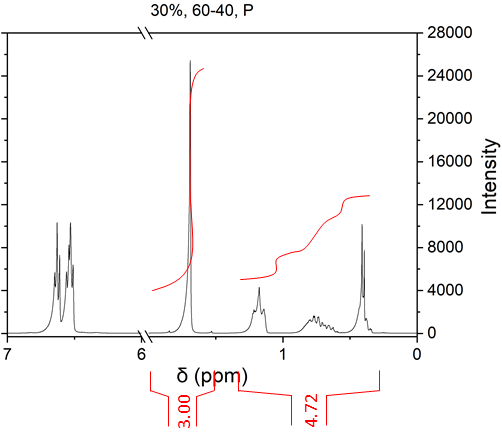
**

b)

**
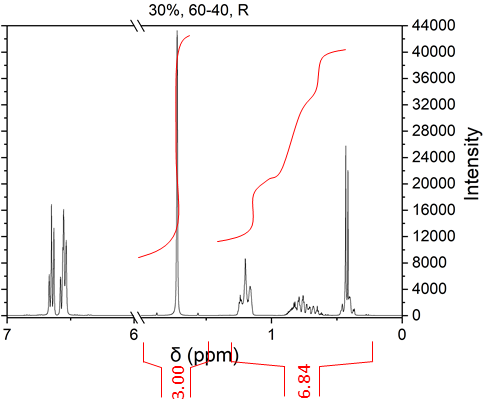
**

c)

Figure S17. ^1^H-NMR spectrum of the feed, permeate and retentate with 30 wt. % 80/20 UiO-66-NH_2_/PdAC in CAS8 membrane, Tol/MCH ~ 60/40 molar ratio. a) feed, b) permeate and c) retentate. It should be noted that the resonance peak at ~1.8 ppm corresponds to the hydrogens in Tol and the resonance peaks at ~0.3-1.4 ppm correspond to the hydrogens in MCH.

**
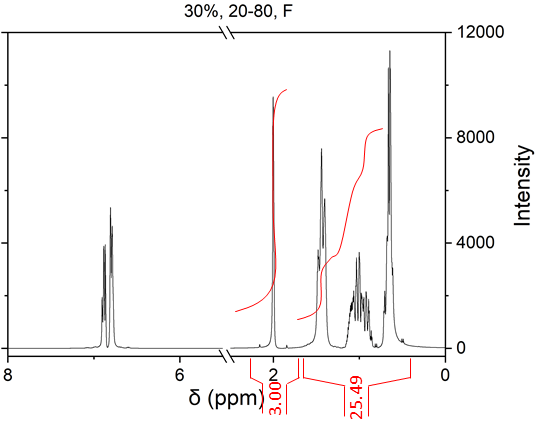
**

a)

**
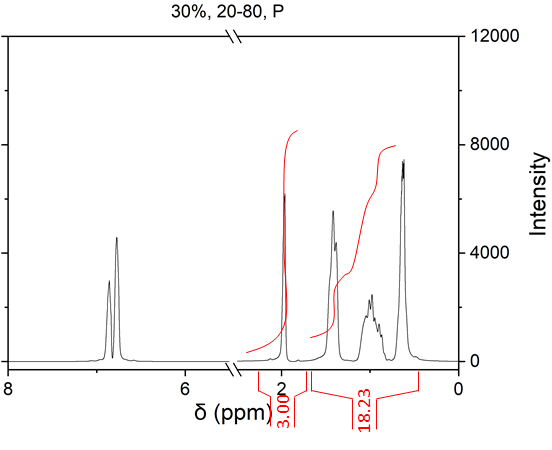
**

b)

**
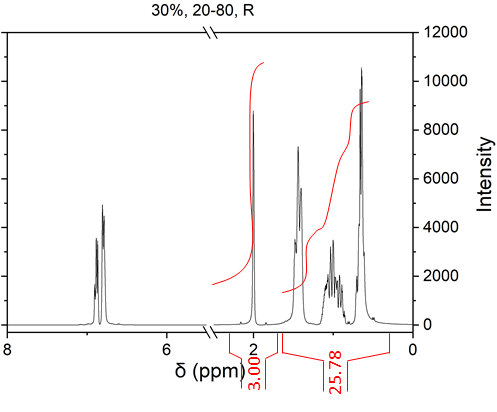
**

c)

Figure S18. ^1^H-NMR spectrum of the feed, permeate and retentate with 30 wt. % 80/20 UiO-66-NH_2_/PdAC in CAS8 membrane, Tol/MCH ~ 20/80 molar ratio. a) feed, b) permeate and c) retentate. It should be noted that the resonance peak at ~2.1 ppm corresponds to the hydrogens in Tol and the resonance peaks at ~0.5-1.8 ppm correspond to the hydrogens in MCH.

**
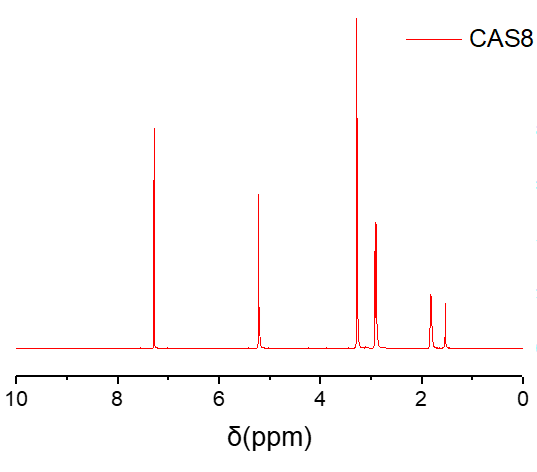
**

Figure S19. 1H-NMR spectrum of CAS8.
